# Pauli Exclusion by n→π* Interactions: Implications for Paleobiology

**DOI:** 10.1021/acscentsci.4c00971

**Published:** 2024-09-04

**Authors:** Jinyi Yang, Volga Kojasoy, Gerard J. Porter, Ronald T. Raines

**Affiliations:** Department of Chemistry, Massachusetts Institute of Technology, Cambridge, Massachusetts 02139, United States

## Abstract

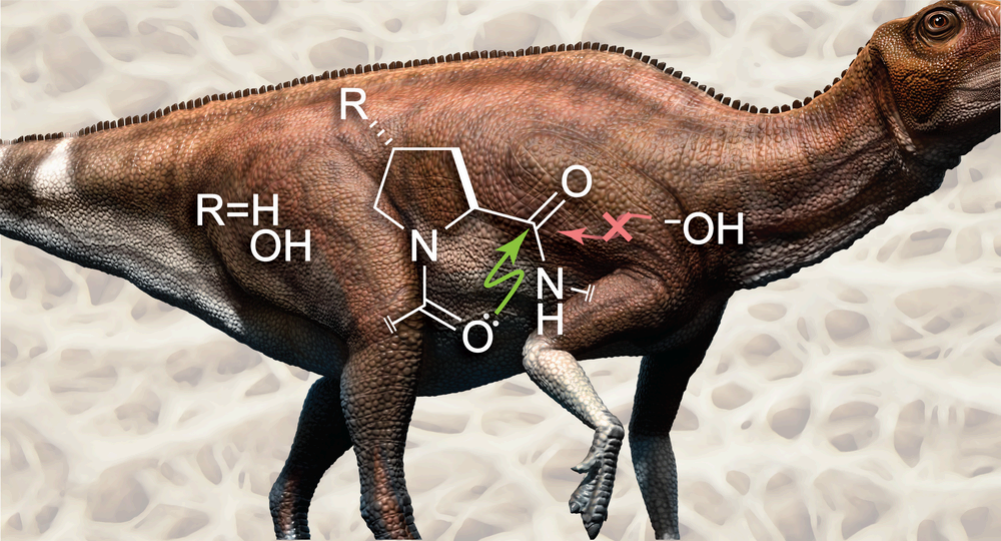

Proteins have evolved to function in an aqueous environment. Collagen, which provides the bodily scaffold for animals, has a special need to retain its integrity. This need was addressed early on, as intact collagen has been detected in dinosaur fossils, even though peptide bonds have a half-life of only ∼500 years in a neutral aqueous solution. We sought to discover the physicochemical basis for this remarkable resistance to hydrolysis. Using experimental and computational methods, we found that a main-chain acyl group can be protected from hydrolysis by an O···C=O n→π* interaction with a neighboring acyl group. These interactions engage virtually every peptide bond in a collagen triple helix. This protection, which arises from the Pauli exclusion principle, could underlie the preservation of ancient collagen.

## Abstract 2

Dinosaur collagen has survived, despite peptides being stable for only centuries. We found that electron donation from an adjacent peptide bond stabilizes each peptide bond within a collagen helix.

## Introduction

Collagen is the most abundant protein in modern animals. Bone collagen is commonly used for paleobiological dating because of its deep roots in the kingdom Animalia.^1^ For example, fragments of type-I collagen have been extracted from the bones of 68 and 80 million-year-old fossils of *Tyrannosaurus rex* and *Brachylophosaurus canadensis*, respectively.^2,3^ More recently, Gly-Pro-Hyp (where Hyp is (2*S*,4*R*)-4-hydroxyproline), which is the most abundant tripeptide sequence in collagen, has been detected in a rib of a 195 million-year-old *Lufengosaurus*.^4^ This longevity is surprising because a peptide bond has a half-life of only ∼500 years in a neutral aqueous solution at 25 °C.^5−8^

Numerous factors have been proposed for the extraordinary longevity of collagen. The abundance of the protein, its highly cross-linked structure, and its inaccessibility to proteases could play roles.^9^ In addition, the mineral matrix within bones could deter the extraction of collagen and its subsequent exposure to hydrolytic conditions.^10^ None of these putative explanations is definitive, and none provides a physicochemical basis for the resistance of the peptide bonds in collagen to hydrolysis.

Main-chain O*_i_*_–1_···C′*_i_*=O_*i*_ n→π* interactions are important for the stability of proteins.^11−15^ This electronic interaction has *E*_n→π*_ = 0.3–0.7 kcal/mol and is highly abundant, especially in collagen.^15,16^ Each strand in the collagen triple helix adopts a polyproline II-type (PPII) helix.^17,18^ In a PPII helix, adjacent carbonyl groups are engaged in an n→π* interaction.^19^ These interactions provide electron density to 99.9% of the carbonyl π* orbitals in natural collagen.^20^ These same π* orbitals are the ones vulnerable to attack by water during hydrolysis. The ensuing competition arises from the Pauli exclusion principle, which opposes the “crowding” of filled orbitals.^21,22^ Accordingly, the engagement of a π* orbital in an n→π* interaction could protect peptide bonds from hydrolysis. Herein, we test this hypothesis.

## Results and Discussion

### Experimental Design

**Scheme 1:**
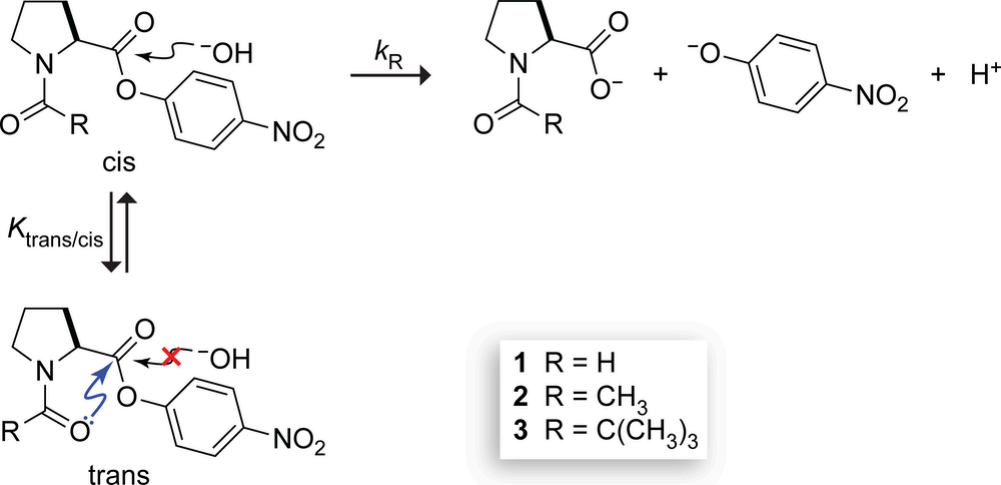
Assay to Detect Pauli Exclusion by an n→π* Interaction

We used a molecular “torsion balance” to test our hypothesis. Approximately 1/4 of the residues in collagen are Pro or Hyp.^23−25^ Their pyrrolidine rings constrain the main-chain *ϕ* (C′_*i*–1_–N_*i*_–C^α^_*i*_–C′_*i*_) dihedral angle to be close to that of an ideal PPII helix. Prolyl peptide bonds can readily adopt either a trans (*Z*) or a cis (*E*) isomerization state.^26,27^ An n→π* interaction is possible only with trans peptide bonds, like those in a PPII helix. Accordingly, the cis–trans equilibrium constant (*K*_trans/cis_ = [trans]/[cis]) correlates with the strength of an n→π* interaction.^15,28^ As a model system, we chose *N*-acylated (2*S*)-proline *p*-nitrophenyl (pNP) esters. We reasoned that the hydrolysis rate of a *p*-nitrophenyl ester, monitored by the release of *p*-nitrophenolate, can serve as a proxy for understanding the hydrolytic stability of a peptide bond (Scheme 1). The use of an ester avoids the complications of γ-turn formation, as has been observed in both *N*-acetyl proline *N*-methylamide and *N*-pivaloyl proline *N*-methylamide.^29−31^ By *N*-acylating proline *p*-nitrophenyl esters with acyl groups of varying size, we sought to use a steric clash to modulate *K*_trans/cis_ and thus the prevalence of an n→π* interaction.^13,32−36^ In our experimental design, the n→π* (donor–acceptor) stabilizations would be subject to experimental modulation by altering the steric (donor–donor) repulsions of surrounding pendant groups. As shown by natural steric analysis,^37^ such steric repulsions can also be traced to the constraints imposed by the Pauli exclusion principle.^21,22^

### Reaction Kinetics

**Figure 1:**
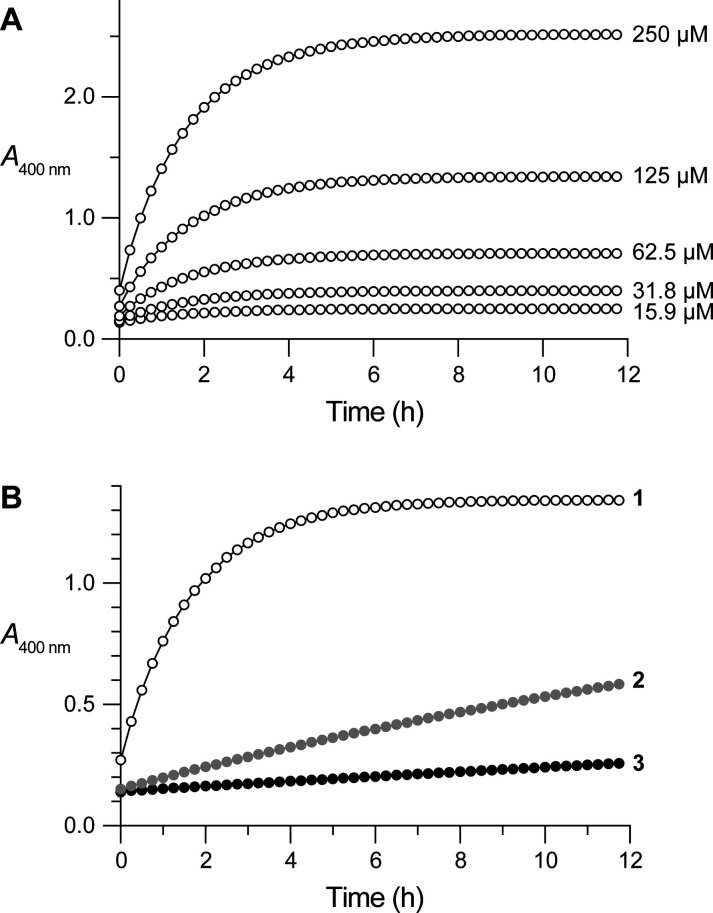
Graphs showing the time course for the spontaneous hydrolysis of *N*-acylated (2*S*)-proline *p*-nitrophenyl esters in 20 mM Tris–HCl buffer, pH 8.0, containing NaCl (0.10 M) and DMSO (1% v/v). (A) Ester **1** at various concentrations. (B) Esters **1**–**3** at 125 μM (including the data for ester **1** from panel A).

We synthesized (2*S*)-proline *p*-nitrophenyl esters that were *N*-acylated with a formyl (**1**), acetyl (**2**), or pivaloyl (**3**) group (Scheme 1; Figures S1–S3). We found that, as expected,^13,32−36^ greater steric bulk gave rise to a higher value of *K*_trans/cis_ (Table 1). Next, we monitored the spontaneous hydrolysis of each ester (**1**–**3**) in Tris–HCl buffer, pH 8.0, containing NaCl (0.10 M) and DMSO (1% v/v) based on the absorbance of the *p*-nitrophenolate product at 400 nm (Figures S6 and S7).^38^ As expected, increasing the ester concentration led to the faster production of *p*-nitrophenolate (Figures 1A and S7).

**Table 1:**
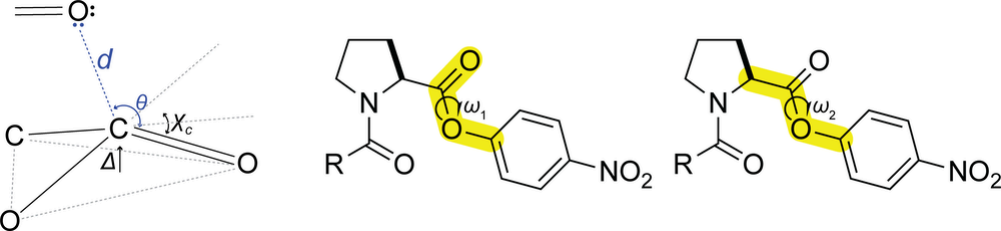
Thermodynamic and Kinetic Parameters of *N-*Acylated (2*S*)-Proline *p*-Nitrophenyl Esters **1**–**3**

prolyl ester	*K*_trans/cis_^a^	*k*_obs_ (10^–6^ s^–1^)^b^	*k*_R_ (10^–6^ s^–1^)^c^
FmProOpNP (**1**)	2.4 ± 0.1	148 ± 2	503 ± 16
AcProOpNP (**2**)	8.8 ± 0.2	13.7 ± 0.6	134 ± 6
PivProOpNP (**3**)	≥20	2.9 ± 0.1	≥61

a Values are from ^1^H NMR spectroscopy in 20 mM sodium phosphate buffer, pH 8.0, containing NaCl (0.10 M) and DMSO (1% v/v).

b Values are from the initial rate data in Figure 1B and eq 1: d[*p*-nitrophenolate]/d*t* = *k*_obs_[ester].

c Values are from eq 5: *k*_R_ = *k*_obs_(1 + *K*_trans/cis_).

Importantly, we observed a correlation between the trans/cis ratio and the hydrolysis rate of the esters: the higher the *K*_trans/cis_, the lower the pseudo-first-order rate constant, *k*_obs_ (Table 1). Ester **1**, which is ∼29% in the cis isomerization state, hydrolyzed rapidly (Figure 1). In contrast, ester **3**, which is <5% in the cis state, had the slowest hydrolysis rate (Figure 1B). Compound **2** was intermediate in both attributes.

In the mechanism of Scheme 1, *p*-nitrophenolate is produced only from the cis isomer. If so, then the value of *k*_R_ can be calculated from the values of *k*_obs_ and *K*_trans/cis_, assuming that the trans–cis equilibration is relatively rapid. This assumption is valid based on known rates of prolyl peptide bond isomerization. For example, the rate constants for the isomerization of *N*-acetyl (2*S*)-proline methyl ester are 0.010 s^–1^ (trans → cis) and 0.024 s^–1^ (cis → trans) in water at 37 °C.^39^ These values are much larger than those of *k*_obs_ (Table 1). Moreover, none of the time courses exhibited the “burst” kinetics characteristic of slow trans–cis isomerization (Figures 1A and S7).^40^ Accordingly, *k*_obs_, *k*_R_, and *K*_trans/cis_ of Scheme 1 are related as follows.

The rate of hydrolysis is
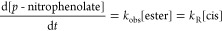
1

The conservation of mass requires that
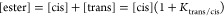
2and rearranging gives
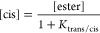
3

Combining eq 1 and eq 3 yields
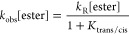
4and rearranging gives
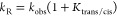
5

**Figure 2:**
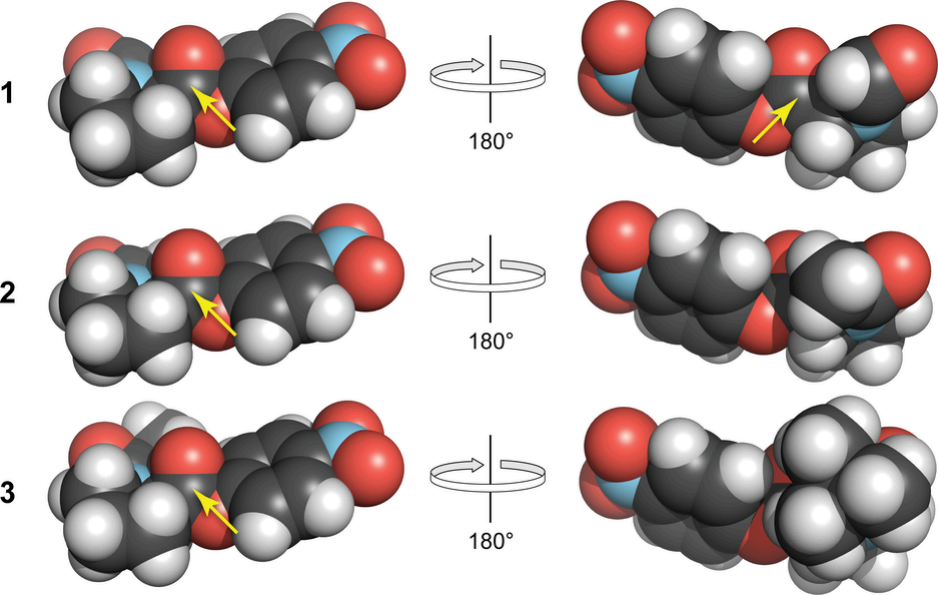
Energy-minimized structures of the cis isomers of *N*-acylated (2*S*)-proline *p*-nitrophenyl esters **1**–**3**. These isomers lack protective n→π* interactions. The yellow arrows point to solvent-accessible ester carbons that could be attacked by a water molecule during a hydrolysis reaction, as in Scheme 1. The image was made with PyMOL software.^42^

**Figure 3:**
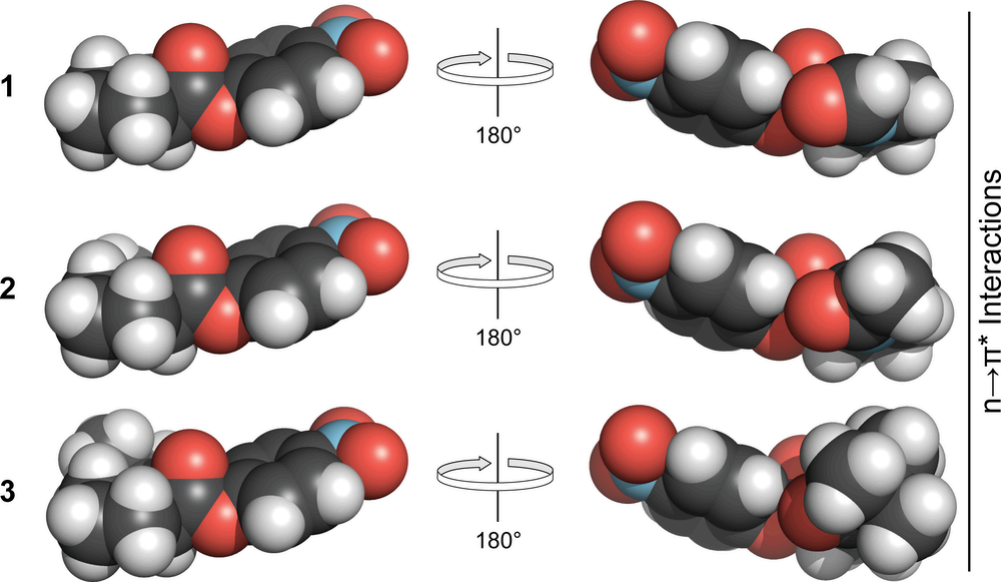
Energy-minimized structures of the trans isomers of *N*-acylated (2*S*)-proline *p*-nitrophenyl esters **1**–**3**. These isomers have n→π* interactions that could protect their solvent-accessible ester carbon from attack by a water molecule by Pauli exclusion. The image was made with PyMOL software.^42^

The values of *k*_R_ calculated with eq 5 are listed in Table 1. These values are similar to those reported for the hydrolysis of *p*-nitrophenyl acetate under comparable conditions.^41^ The variance of the *k*_R_ values is small, consistent with the mechanism of Scheme 1 and indicative of similar reactivity for the cis isomers of esters **1**–**3**. The small differences in *k*_R_ values likely arise from a steric effect on hydrolysis in the cis isomer. For example, when R = H, the ester carbon in the cis isomer is more accessible to attack by water than when R = Me or *t*-Bu (Figures 2 and S9). Though accessible in the trans isomers, the ester carbons of esters **1**–**3** would be protected from attack by Pauli exclusion in that isomerization state (Figure 3). This interpretation is consistent with an observed depletion—by hydrolysis—of the cis isomer at a low temperature (Figure S8), where the trans → cis isomerization of prolyl peptide bonds is slow.^43^ Altogether, the evidence supports our hypothesis that an n→π* interaction deters hydrolysis, as in Scheme 1.^44,45^

### Computational Analyses

**Figure 4:**
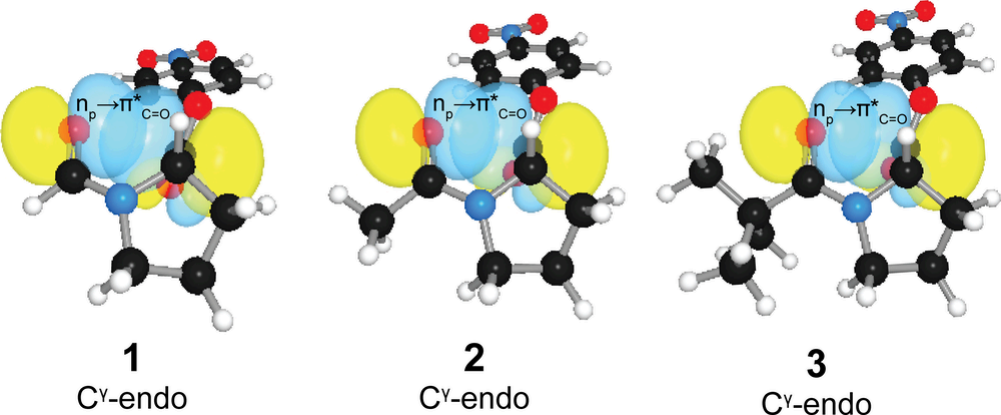
Images of the overlap between the amide oxygen lone pair in a p-rich orbital (n_p_) and the antibonding orbital (π*_C=O_) of the ester carbonyl group in energy-minimized structures of *N*-acylated (2*S*)-proline *p*-nitrophenyl esters **1**–**3** with a C^γ^-endo ring pucker. Overlap integrals: **1**, 0.1281; **2**, 0.1327; and **3**, 0.1451. Depictions were generated with the program NBOPro7@Jmol.^52^

To gain further insight into the stabilization afforded by n→π* interactions,^46,47^ we performed a series of computational analyses^33,34,48,49^ using density functional theory (DFT) at the M06-2X/6-31+G(d,p) level of theory.^50,51^ To validate the robustness of our chosen level of theory, we performed benchmark analyses with different methods (see: Tables S1–S6). Natural bond orbital (NBO) calculations were carried out with NBO v7.0 implemented in Gaussian 16.^52,53^ We found that in the preferred conformation of ester **3**, the O_*i*–1_···C_*i*_ distance is *d* = 2.628 Å (cf., the sum of the van der Waals radii, which is *r*_W_(O) + *r*_W_(C) = 3.22 Å) and the O_*i*–1_···C_*i*_=O_*i*_ angle is *θ* = 98.88°, which aligns with the Bürgi–Dunitz trajectory.^54−56^ The carbonyl carbon is at the apex of a pyramid with *Δ* = 0.020 Å and *χ*_C_ = 2.81°. To characterize the n→π* interaction, we calculated the corresponding second-order perturbation energies above a 0.1 kcal/mol threshold. The overlap increased in the order **1** < **2** < **3** (Figure 4). The trend in *E*_n→π*_ (**1** < **2** < **3**) was maintained in esters with the same pyrrolidine ring pucker (i.e., C^γ^-exo and C^γ^-endo) in vacuo (Table S7) and with implicit water solvation (Table S8).

### X-ray Crystallography

**Figure 5:**
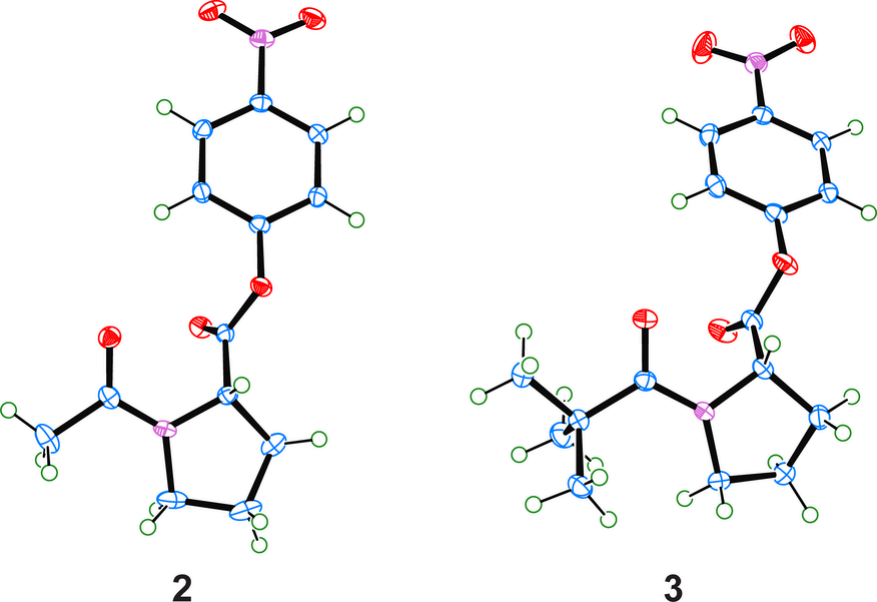
ORTEP of esters **2** and **3** from X-ray crystallography.

To validate our computational analyses, we obtained the X-ray crystal structure of esters **2** and **3** (Figure 5). These experimental structures (e.g., bond lengths and bond angles) are similar to those in the computationally optimized structures having the same pyrrolidine ring pucker (i.e., C^γ^-exo or C^γ^-endo) (Table S9). Likewise, the angles and distances related to the n→π* interaction are in gratifying agreement with those calculated with DFT (Table 2) and confirm a strong n→π* interaction. In addition, experimental NMR chemical shifts align well with those calculated for esters **1**–**3** (Tables S10–S13).^58^

**Table 2:**
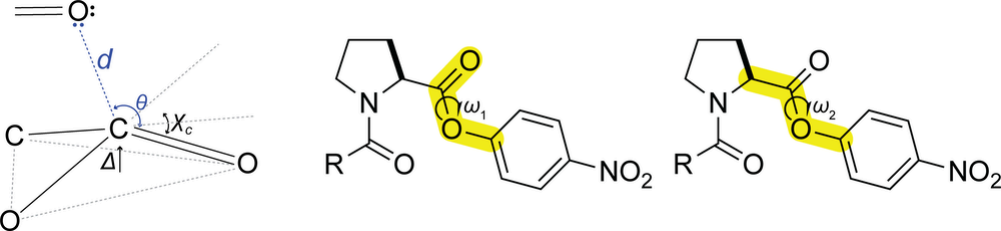
Energetic and Structural Parameters of the Trans Isomer of *N*-Acylated (2*S*)-Proline *p*-Nitrophenyl Esters **1**–**3**

	computational^a^			experimental^b^	
	**1**	**2**	**3**	**2**	**3**
parameter	C^γ^-endo	C^γ^-exo	C^γ^-endo	C^γ^-exo	C^γ^-endo
Electronic					
*E*_n→π*_ (kcal/mol)	2.53	3.66	3.39	—	—
π* Occupancy	0.1845	0.1876	0.1876	—	—
Bürgi–Dunitz					
*d* (Å)	2.724	2.699	2.628	2.730	2.720
*θ* (deg)	97.13	98.29	98.88	95.80	97.30
Pyramidylization^c^					
*Δ* (Å)	0.016	0.018	0.020	0.032	0.021
*χ*_C_ (deg)	2.30	2.62	2.81	4.51	2.99

a Optimized geometries, energies, and occupancies were calculated with M06-2X/6-31+G(d,p). *E*_n→π*_ values are contributions from p-type lone pairs as calculated with second-order perturbation theory. Pyrrolidine ring puckers (i.e., C^γ^-exo and C^γ^-endo) were chosen to correspond to those in the experimental structures.

b Values were determined by X-ray diffraction analysis (Figure 5).

c Values of *Δ* were determined with Mercury software (CDCC). *χ*_C_ = *ω*_1_ – *ω*_2_ + π(mod 2π).^57^

### n→π* Interactions in the Collagen Triple Helix

**Figure 6:**
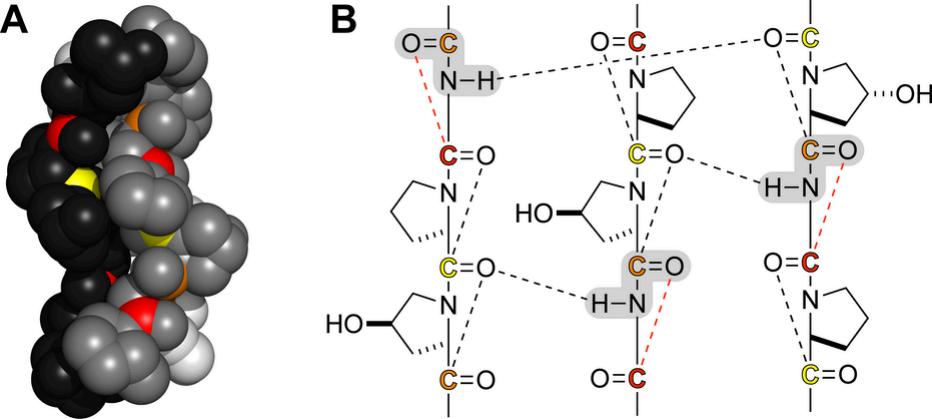
Interactions in a collagen triple helix. (A) Crystal structure of a (Pro-Hyp-Gly)_*n*_ triple helix. The three strands are white, gray, or black. In each strand, peptide-bond carbons are yellow (Pro-Hyp), orange (Hyp-Gly), or red (Gly-Pro). The image was made with PyMOL software^42^ and PDB entry 1v7h.^59^ (B) n→π* Interactions and hydrogen bonds within the main chain of a collagen triple helix. Carbons are colored as in panel A. The shaded peptide bonds are polarized by interstrand hydrogen bonds.

There are three types of peptide bonds in a collagen triple helix: Xaa-Yaa, Yaa-Gly, and Gly-Xaa. The N–H group of a Yaa-Gly peptide bond donates a hydrogen bond to the O=C group of an Xaa-Yaa peptide bond on another strand. These _Gly_N–H···O=C_Xaa_ hydrogen bonds are strong—each contributes Δ*G*° = 2.0 kcal/mol to the stability of the triple helix.^60^ Their location near the center of the helical axis renders the Xaa-Yaa and Yaa-Gly peptide bonds inaccessible to solvent water (Figure 6A). In comparison, the Gly-Xaa peptide bond is more accessible and thus vulnerable to attack by solvent water. The Gly-Xaa peptide bond is, however, the acceptor of an n→π* interaction from the oxygen of the polarized Yaa-Gly peptide bond (Figure 6B). Thus, the “weakest links” in the collagen triple helix could be most protected by n→π* interactions.

## Conclusions

Using both experimental and computational tools, we have discovered that n→π* interactions protect prolyl esters from hydrolysis. This discovery has implications for the stability of collagen, which is replete with n→π* interactions and has remained intact for (at least) hundreds of millions of years, exceeding the half-life of a peptide bond by a millionfold or more. The stability conferred by n→π* interactions upon collagen—modern and ancient—can guide the design of exceptionally stable, long-lived materials.

## Supporting Information Available

Procedures for the synthesis and analysis of esters **1**–**3**, additional experimental and computational data, Tables S1–S35, and Figures S1–S14 (PDF)
