# Supplementary material for: Pauli Exclusion by n→π* Interactions: Implications for Paleobiology
Source: ACS Cent Sci. 2024 Sep 4;10(10):1829–34. doi: 10.1021/acscentsci.4c00971 (PMC11503490; doi:10.1021/acscentsci.4c00971)
Supplement: Supplementary file 1 — oc4c00971_si_001.pdf [file oc4c00971_si_001.pdf]

# Pauli Exclusion by n→π* Interactions: Implications for Paleobiology

Jinyi Yang, Volga Kojasoy, Gerard J. Porter, and Ronald T. Raines*
Department of Chemistry, Massachusetts Institute of Technology, Cambridge, Massachusetts 02139, United States

## Table of Contents

**Table of Contents** S1

**Synthesis of Proline Esters** S2
General Experimental S2
*N*-Formyl-(2*S*)-proline (**S1**) S3
*N*-Pivaloyl-(2*S*)-proline (**S2**) S3
General procedure for the synthesis of proline nitrophenol esters S4
*N*-Formyl-(2*S*)-proline *p*-nitrophenyl ester (**1**) S4
*N*-Acetyl-(2*S*)-proline *p*-nitrophenyl ester (**2**) S4
*N*-Pivaloyl-(2*S*)-proline *p*-nitrophenyl ester (**3**) S5
*N*-Formyl-(2*S*)-proline methyl ester S6
*N*-Pivaloyl-(2*S*)-proline methyl ester S6

**Assays of Ester Hydrolysis** S7

**Computational Studies** S9
Benchmark studies on esters **1**–**3** (Tables S1–S6) S10
Computational parameters of esters **1**–**3** in vacuo (Table S7) S13
Computational parameters of esters **1**–**3** in water (Table S8) S13
Energies, frequencies, and coordinates of computed structures S13
Computational analysis of nucleophilic attack on cis isomers of esters **1**–**3** (Figure S9) S14
Key bond lengths and angles in esters **1**–**3** (Table S9) S14
Computational NMR data for esters **1**–**3** (Tables S10–S13) S15

**X-Ray Crystal Structures** S19
Crystal data and structure refinement for ester **2** (Tables S14–S18; Figure S10) S19
Crystal data and structure refinement for ester **3** (Tables S19–S24; Figures S11 and S12) S26
Crystal data and structure refinement for *N*-pivaloyl-(2*S*)-proline methyl ester (Tables S25–S29; Figure S13) S34
Crystal data and structure refinement for acid **DS2** (Tables S30–S35; Figure S14) S41

**NMR Spectra** S56
**FTIR Spectra** S63
**References** S64

## Synthesis of Proline Esters

**General Experimental.** All synthetic procedures were performed at ambient temperature (~22 °C) and pressure (~1.0 atm) unless indicated otherwise. All reactions were performed in a reaction vial fitted with TFE-silicone septa under $N_2$(g) using standard Schlenk-line techniques. Reactions carried out at low temperatures were cooled by cooling agents in a Dewar vessel (water-ice bath at 0 °C).

Commercial chemicals were of reagent grade or better from Sigma–Aldrich (St. Louis, MO) and were used without further purification unless indicated otherwise. In all reactions involving anhydrous solvents, glassware was either oven- or flame-dried. Reagent-grade dichloromethane (DCM) was dried over a column of alumina and removed from a dry still under an inert atmosphere. All reactions were magnetically stirred and monitored by liquid chromatography–mass spectrometry (LC–MS) and analytical thin-layer chromatography (TLC). Purification was done with flash column chromatography performed with silica gel or a Biotage Isolera One system unless indicated otherwise. The term "concentrated under reduced pressure" refers to the removal of solvents and other volatile materials using a Buchi rotary evaporator (model R-210) at water aspirator pressure (<20 torr) while maintaining the water-bath temperature below 40 °C.

$^{1}$H and $^{13}$C NMR spectra were acquired with a Bruker (Billerica, MA) Avance Neo 500 MHz spectrometer in the Department of Chemistry Instrumentation Facility (DCIF) at MIT. Proton chemical shifts are reported in parts per million (ppm, $\delta$ scale) and are relative to residual protons in the deuterated solvent ($CDCl_3$: $\delta$ 7.26). Carbon chemical shifts are reported in parts per million (ppm, δ scale) and are relative to the carbon resonance of the solvent ($CDCl_3$: $\delta$ 77.2). For NMR spectra obtained in aqueous solvent, $^{1}$H spectrum was referenced with the 3-(trimethylsilyl)propionic-2,2,3,3-*d*$_4$ acid sodium salt (TSP-*d*$_4$) $^{1}$H resonance. The $^{13}$C spectrum was referenced with the internal TSP $^{1}$H resonance following IUPAC recommendations. $CDCl_3$, $D_2O$, and TSP-*d*$_4$ were from Sigma–Aldrich (Milwaukee, WI). Deuterated phosphate-buffered saline (dPBS) was prepared by dissolving one tablet (Sigma–Aldrich product #P4417) in 200 mL $D_2O$ to obtain 10 mM buffer, pH 7.4, containing NaCl (137 mM) and KCl (2.7 mM). Multiplicities are abbreviated as s (singlet), d (doublet), t (triplet), q (quartet), sept (septet), and m (multiplet). Compounds containing the *N*-formyl-(2*S*)-prolyl moiety and *N*-acetyl-(2*S*)-prolyl moiety exist as mixtures of *Z* and *E* isomers that do not interconvert on the NMR time scale. Accordingly, these compounds exhibit two sets of NMR signals. In $^{13}$C data, signals that clearly arise from the minor *E* isomer are listed within parentheses.

Fourier-transform infrared spectroscopy (FTIR) was performed with a Bruker Alpha II FTIR spectrometer with a Diamond Crystal attenuated total reflectance accessory in the DCIF at MIT. Data were analyzed using the Opus 7.8 software.

Mass spectrometry was performed with an LCT electrospray ionization (ESI) 1260 Infinity II instrument from Agilent Technologies (Santa Clara, CA) and an LC–MS column (Agilent Technologies, Poroshell 120, SB C18-reversed-phase, length 50 mm, internal diameter: 2.1 mm, particle size: 2.7 micron) with a gradient of 10−95% v/v MeCN (0.1% v/v formic acid) in water (0.1% v/v formic acid) over 10 min. High-resolution mass spectrometry (HRMS) was performed with a JEOL AccuTOF 4G LC-plus equipped with an intense DART (Direct Analysis in Real Time) source.

**Safety.** No unexpected or unusually high safety hazards were encountered during the reported work.

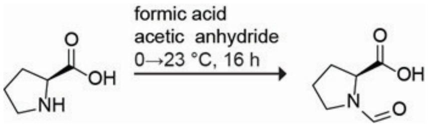


***N*-Formyl-(2*S*)-proline** (**S1**). was prepared as described previously.[1] Briefly, (2*S*)-proline (1.0 g, 8.7 mmol) was dissolved in formic acid (25 mL), and the resulting solution was cooled to 0 °C. The cooled solution was added to a mixture of precooled acetic anhydride (10 mL, 109 mmol) in formic acid (25 mL). After full dissolution, the ice bath was removed, and the reaction mixture was stirred overnight. Solvent was removed under reduced pressure, and the residue was purified by flash chromatography (25 g silica gel, 5% v/v methanol in DCM) to yield a clear oil.

Yield: 80%
R*f* (9:1 DCM/MeOH): 0.41
$^1$H NMR ($CDCl_3$, 500 MHz, $\delta$): 1.90–2.10 (m, 2H), 2.21–2.32 (m, 2H), 3.54–3.68 (m, 2H), 4.44–4.53 (m, 1H), 8.31 and 8.29 (s, 1H)
$^{13}$C NMR ($CDCl_3$, 126 MHz, $\delta$): 24.15 (22.94), 28.67 (29.83), 47.20 (44.37), 57.55 (58.95), 162.64 (162.78), 173.34 (175.12)
MS–ESI (*m*/*z*): $[M + H]^+$ calcd for $C_6H_{10}NO_3$, 144.06; found, 144.0
The characterization data matched those reported in the literature.[1]

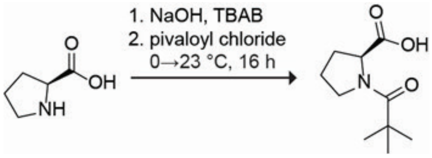


***N*-Pivaloyl-(2*S*)-proline** (**S2**) was prepared as described previously.[2] Briefly, (2*S*)-proline (1.0 g, 8.7 mmol, 1 equiv) was dissolved in water at 0 °C. An aqueous solution of NaOH (0.7 g in 2.5 mL distilled water) was added slowly, followed by the addition of TBAB (0.14 g, 0.43 mmol, 0.05 equiv). The reaction mixture was allowed to stir for 1 h before a solution of pivaloyl chloride (0.97 mL, 0.43 mmol, 0.05 equiv) in DCM was added slowly and stirred for an additional 1 h. Then, the ice water bath was removed, and the reaction mixture was stirred at 25 °C overnight. The aqueous layer was washed with DCM (3 × 25 mL), diluted with 2 N HCl, and the product was extracted with DCM (3 × 25 mL). The organic layer was combined and washed with brine (30 mL), dried over $MgSO_4$(s), filtered, and concentrated under reduced pressure. The product **S2** was recrystallized in EtOAc and used without further purification.

Yield: 42%
R*f* (9:1 DCM/MeOH): 0.52
$^1$H NMR ($CDCl_3$, 500 MHz, $\delta$): 1.29 (s, 9H), 1.93–2.12 (m, 3H), 2.21–2.27 (m, 1H), 3.70–3.73 (m, 2H), 4.59–4.61 (dd, 1H)
$^{13}$C NMR ($CDCl_3$, 126 MHz, $\delta$): 26.01, 26.76, 27.38, 39.27, 48.70, 61.79, 174.49, 179.48
MS–ESI (*m*/*z*): $[M + H]^+$ calcd for $C_{10}H_{18}NO_3$, 200.12; found, 200.1
The characterization data matched those reported in the literature.[2]

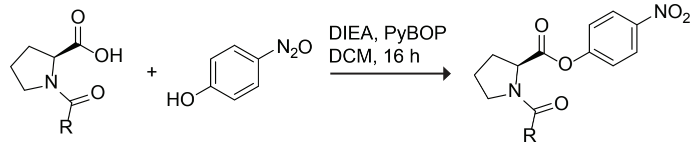


**General procedure for the synthesis of proline nitrophenol esters.** To a solution of *N*-acylated (2*S*)-proline (2.1 mmol) in dry DCM (30 mL) under $N_2$(g), was added *p*-nitrophenol (0.31 g, 2.2 mmol), DIEA (0.7 mL, 4 mmol), and PyBOP (1.09 g, 2.1 mmol). After stirring overnight, the reaction mixture was washed with brine (30 mL). The aqueous layer was extracted with DCM (3 × 25 mL), and all organic layers were combined and washed with 2 N HCl(aq) (3 × 25 mL), saturated $NaHCO_3$(aq) (3 × 25 mL), brine (25 mL), and water (25 mL). The organic layer was dried over $MgSO_4$(s), filtered, and concentrated under reduced pressure.

***N*-Formyl-(2*S*)-proline *p*-nitrophenyl ester (1).** Flash chromatography (4:3:1 hexanes/EtOAc/EtOH) afforded **1** as a colorless oil.

Yield: 24%
R*f* (hexanes/EtOAc/EtOH 4:3:1): 0.34
$^1$H NMR ($CDCl_3$, 500 MHz, $\delta$): 2.00–2.24 (m, 3H), 2.39–2.50 (m, 1H), 3.59–3.78 (m, 2H), 4.63–4.72 (m, 1H), 7.30–7.34 (m, 2H), 8.25–8.31 (m, 2H), 8.36 and 8.40 (s, 1H)
$^{13}$C NMR ($CDCl_3$, 126 MHz, $\delta$): 24.47 (23.04), 29.56 (29.95), 46.51 (44.28), 56.94 (58.95), 122.52 (122.20),125.38 (125.53), 145.68 (145.84), 155.38 (154.87), 161.12 (161.61), 169.64 (169.58)
FTIR (film, cm$^{-1}$): 1012, 1031, 1088, 1127, 1205, 1344, 1378, 1416, 1489, 1520, 1591, 1615, 1659, 1763, 2861, 2882, 2957, 2981, 3081, 3115
HRMS–ESI (*m*/*z*): $[M + H]^+$ calcd for $C_{12}H_{13}N_2O_5$, 265.08190; found, 265.08320
The characterization data matched those reported in the literature.[3]

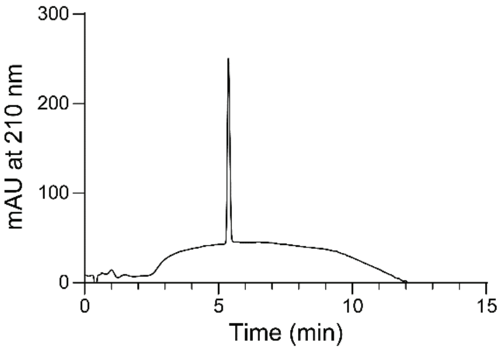


**Figure S1**. LC trace of **1**. RT, 5.38 min.

***N*-Acetyl-(2*S*)-proline *p*-nitrophenyl ester (2).** Flash chromatography (hexanes/EtOAc/EtOH 4:3:1) afforded **2** as a colorless oil.

Yield: 61%
R*f* (4:3:1 hexanes/EtOAc/EtOH): 0.28
$^1$H NMR ($CDCl_3$, 500 MHz, $\delta$): 2.15 (s, 3H), 2.03–2.24 (m, 3H), 2.36–2.44 (m, 1H), 3.58–3.63 (m, 1H), 3.70–3.74 (m, 1H), 4.64–4.66 (m, 1H), 7.29–7.34 (m, 2H), 8.24–8.31 (m, 2H)

$^{13}$C NMR ($CDCl_3$, 126 MHz, $\delta$): 22.25 (23.04), 25.29, 29.58 (31.80), 47.99 (46.67), 59.03 (60.36), 122.57 (122.17), 125.35 (125.58), 145.60, 155.61, 170.07, 170.41

FTIR (film, cm$^{-1}$): 1012, 1127, 1182, 1204, 1255, 1344, 1415, 1489, 1521, 1591, 1615, 1637, 1765, 2879, 2955, 2980, 3083, 3113

HRMS–ESI (*m*/*z*): [M + H]$^+$ calcd for $C_{13}H_{15}N_2O_5$, 279.09755; found, 279.09834

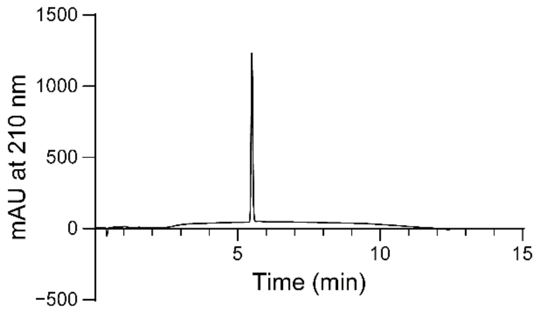


**Figure S2**. LC trace of **2**. RT, 5.50 min.

***N*-Pivaloyl-(2*S*)-proline *p*-nitrophenyl ester (3).** Flash chromatography (4:3:1 hexanes/EtOAc/EtOH) afforded **3** as a colorless oil. **Crystallization conditions.** Ester **3** was subjected to reversed-phase high-performance liquid chromatography (HPLC) using a preparatory or XSelect Peptide CSH C18 column from Waters (Milford, MA), lyophilized as a TFA salt, and redissolved in hexanes containing minimal dichloromethane. Crystals of ester **3** were observed after two weeks at room temperature. Heating was avoided due to the hydrolysis of ester **3**. Heating did lead to the crystallization of *N*-pivaloyl-(2*R*)-proline (**DS2**) during an unsuccessful racemic crystallization experiment with ester **3**. The X-ray structure of acid **DS2** is reported below.

Yield: 21%

R*f* (4:3:1 hexanes/EtOAc/EtOH): 0.48

$^1$H NMR ($CDCl_3$, 500 MHz, $\delta$): 1.29 (s, 9H), 2.03–2.11 (m, 2H), 2.16–2.23 (m, 1H), 2.27–2.34 (m, 1H), 3.77–3.88 (ddt, 2H), 4.65–4.68 (dd, 1H), 7.30–7.33 (m, 2H), 8.24–8.27 (m, 2H)

$^{13}$C NMR ($CDCl_3$, 126 MHz, $\delta$): 26.47, 27.31, 27.86, 38.86, 48.48, 61.42, 122.55, 125.31, 145.48, 155.83, 170.84, 177.46

FTIR (film, cm$^{-1}$): 1013, 1047, 1094, 1121, 1158, 1209, 1344, 1362, 1379, 1406, 1489, 1522, 1592, 1614, 1768, 2878, 2935, 2973, 3083, 3117

HRMS–ESI (*m*/*z*): [M + H]$^+$ calcd for $C_{16}H_{21}N_2O_5$, 321.14450; found, 321.14548

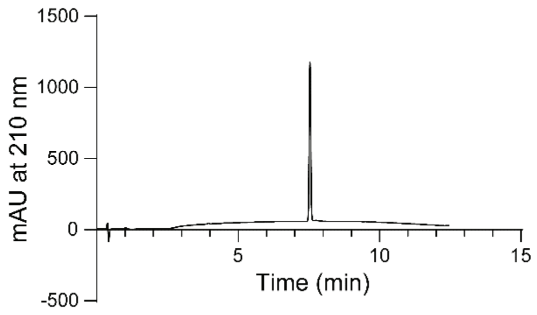


**Figure S3**. LC trace of **3**. RT, 7.54 min.

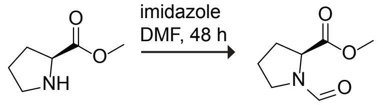


***N*-Formyl-(2*S*)-proline methyl ester** was prepared as described previously.[4] Briefly, (2*S*)-proline methyl ester (165 mg, 1 mmol, 1 equiv) and imidazole (136 mg, 2 mmol, 2 equiv) were dissolved in DMF (1 mL). The reaction mixture was allowed to stir at 60 °C for 48 h. After solvent removal, the residue was dissolved in saturated $NaHCO_3$(aq) (20 mL) and extracted by EtOAc (3 × 20 mL). The organic layer was combined and dried over $MgSO_4$(s), filtered, and concentrated under reduced pressure. Flash chromatography (4:3:1 hexanes/EtOAc/EtOH) afforded *N*-formyl-(2*S*)-proline methyl ester as a yellow oil.

Yield: 50%
R*f* (4:1 DCM/MeOH): 0.33
$^1$H NMR (dPBS, 500 MHz, *δ*): 1.85–2.11 (m, 3H), 2.19–2.42 (m, 1H), 3.44–3.59 (m, 1H), 3.67–3.77 (m, 1H), 3.79 and 3.80 (s, 3H), 4.43–4.75 (m, 1H), 8.19 and 8.24 (s, 1H)
$^{13}$C NMR (dPBS, 126 MHz, *δ*): 23.71 (22.35), 29.22 (29.26), 47.40 (44.29), 53.14 (53.29), 56.99 (59.64), 163.64 (164.76), 174.36 (174.74)
FTIR (film, cm$^{-1}$): 1002, 1035, 1092, 1173, 1381, 1437, 1669, 1736, 2861, 2875, 2930, 2957
HRMS–ESI (*m*/*z*): $[M + H]^+$ calcd for $C_7H_{11}NO_3$, 158.08117; found, 158.08204
The characterization data matched those reported in the literature.[4]

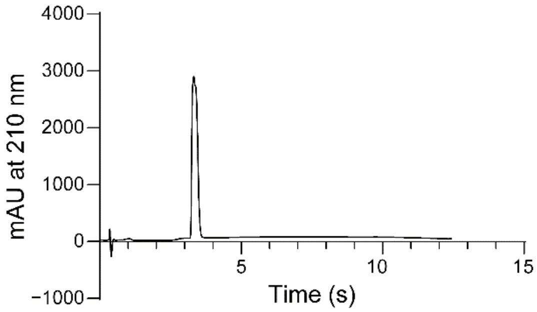


**Figure S4**. LC trace of *N*-formyl-(2*S*)-proline methyl ester. Gradient of 2−95% v/v MeCN (0.1% v/v formic acid) in water (0.1% v/v formic acid) over 10 min. RT, 3.32 and 3.38 min (two isomers).

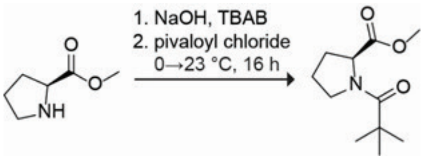


***N*-Pivaloyl-(2*S*)-proline methyl ester** was prepared as described previously.[5] Briefly, (2*S*)-proline methyl ester (1 g, 6.1 mmol, 1 equiv) was dissolved in water at 0 °C. An aqueous solution of NaOH (0.7 g in 2.5 mL of water) was added slowly, followed by the addition of TBAB (0.1 g, 0.3 mmol, 0.05 equiv). The reaction mixture was allowed to stir for 1 h before a solution of pivaloyl chloride (0.7 mL, 0.3 mmol, 0.05 equiv) in DCM was added slowly and stirred for an additional 1 h. Then, the ice water bath was removed, and the reaction mixture was stirred at 25 °C overnight. The aqueous layer was washed with DCM (3 × 25 mL), diluted with 2 N HCl, and the product was extracted with DCM (3 × 25 mL). The organic layer was combined and washed with brine (30 mL), dried over $MgSO_4$(s), filtered, and concentrated under reduced pressure. Flash chromatography (4:3:1 hexanes/EtOAc/EtOH) afforded *N*-pivaloyl-(2*S*)-proline methyl ester as a colorless solid.

Yield: 65%
R*f* (4:1 DCM/MeOH): 0.81
$^{1}$H NMR (dPBS, 500 MHz, *δ*): 1.25 (s, 9H), 1.83–1.90 (m, 1H), 1.96–2.10 (m, 2H), 2.19–2.26 (m, 1H), 3.75 (s, 3H), 3.80–3.90 (m, 2H), 4.40–4.43 (m, 1H)
$^{13}$C NMR (dPBS, 126 MHz, *δ*): 25.70, 26.32, 27.53, 38.57, 48.97, 52.79, 61.77, 175.81, 180.12
FTIR (film, cm$^{-1}$): 1017, 1030. 1050. 1095, 1166, 1192, 1244, 1280, 1361, 1378, 1405, 1435, 1480, 1510, 1563, 1588, 1620, 1744, 2877, 2957, 2972
HRMS–ESI (*m*/*z*): $[M + H]^{+}$ calcd for $C_{11}H_{19}NO_3$, 214.14377; found, 214.14556
The characterization data matched those reported in the literature.[5]

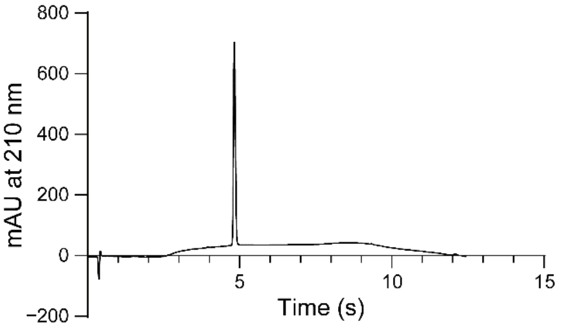


**Figure S5**. LC trace of *N*-pivaloyl-(2*S*)-proline methyl ester. RT, 4.82 min.

### Assays of Ester Hydrolysis

Compound stocks were prepared in anhydrous DMSO then diluted to the desired concentration with a final solvent composition of 1% v/v DMSO in 20 mM Tris–HCl buffer, pH 8.0, containing NaCl (0.10 M). Ester hydrolysis was monitored by measuring the absorbance of the *p*-nitrophenolate product at 400 nm. Samples were prepared in triplicates. At the end of the scan, samples were quenched with 1 M NaOH, and the absorbance at 400 nm was recorded to calibrate the initial concentration using a standard curve generated with known concentrations of *p*-nitrophenol. Initial rates were obtained by fitting the linear range of the hydrolysis scan with linear regression using Prism 10.0.3 (GraphPad Software, Boston, MA) and were plotted against calibrated concentrations to obtain pseudo-first order rate constants.

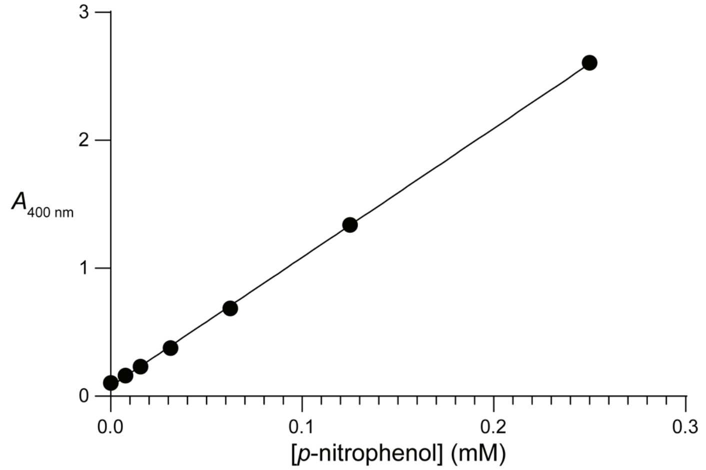


**Figure S6**. Graph showing the concentration-dependence of *p*-nitrophenolate absorbance at 400 nm in 20 mM Tris–HCl buffer, pH 8.0, containing NaCl (0.10 M) and DMSO (1% v/v). Linear regression analysis yields $A_{400\ nm}$ = 10.08 × [*p*-nitrophenol] (mM) + 0.07705.

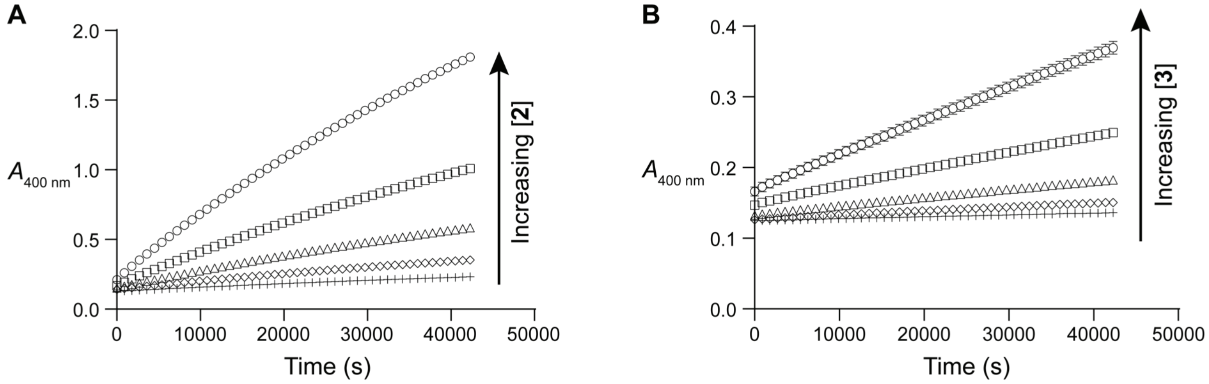


**Figure S7.** Graphs showing the time course for the spontaneous hydrolysis of esters **2** and **3** at various concentrations in 20 mM Tris–HCl buffer, pH 8.0, containing NaCl (0.10 M) and DMSO (1% v/v). (A) [**2**]: 0.028 mM, 0.059 mM, 0.12 mM, 0.24 mM, and 0.48 mM. (B) [**3**]: 0.022 mM, 0.066 mM, 0.12 mM, 0.23 mM, and 0.46 mM.

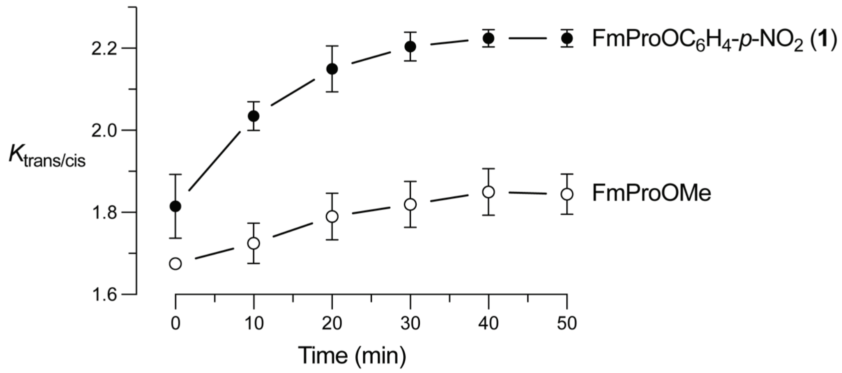


**Figure S8.** Graph showing the change in the values of $K_{trans/cis}$ for ester **1** and FmProOMe over time at low temperature (10 °C) in 20 mM sodium phosphate, pH 8.0, containing NaCl (0.10 M) and DMSO (1% v/v) and monitored by $^1$H NMR spectroscopy. Notably, the first measurement was taken after ~10 min to enable the temperature to stabilize and the tuning of the NMR spectrometer.

## Computational Studies

All geometry optimizations were carried out with Gaussian 16 using density functional theory (DFT) at the M06-2X/6-31+G(d,p) level of theory.[6,7] Benchmark studies with different DFT functionals, basis sets and SMD implicit water solvation model were performed to justify our chosen level of theory (Tables S1–S6). Natural bond orbital (NBO) calculations were performed with NBO, version 7.0 implemented in Gaussian 16.[8,9] Conformational searches were carried out using CREST, version 2.12 using gfn2//gfnff in water and in vacuum.[10,11] All conformers resulting from the conformational search in vacuum were optimized at the M06-2X/3-21G level, and any of those with relative energies within 5 kcal/mol of the lowest energy conformer were reoptimized at the M06-2X/6-31+G(d,p) level of theory. For the conformations with a relative energy within 2 kcal/mol of the lowest energy conformation at the M06-2X/6-31+G(d,p) level, computational NMR calculations were performed with SMD($CHCl_3$)-mPW1PW91/6-311+G(2d,p)//M06-2X/6-31+G(d,p)[6,7,12-14] using the GIAO method.[15-18] All conformers resulting from the conformational search in water were optimized at the SMD($H_2O$)-M06-2X/6-31+G(d,p) level of theory. For the conformations with a relative energy within 2 kcal/mol of the lowest energy conformation at the SMD($H_2O$)-M06-2X/6-31+G(d,p) level, computational NMR calculations were performed with SMD($CHCl_3$)-mPW1PW91/6-311+G(2d,p)// SMD($H_2O$)-M06-2X/6-31+G(d,p)[6,7,12-14] using the GIAO method.[15-18] Computed chemical shifts were calculated using scaling factors ($^{13}$C: slope = −1.0446, intercept = 186.7246; $^1$H: slope = −1.0938, intercept = 31.8723) from the CHESHIRE repository[19] and were weighted using a Boltzmann-weighted average.

**Table S1.** Benchmark study on compound **1** with *endo* ring pucker.

| Method | Sum of Electronic and Thermal Free Energies (Hartree) | Lowest Frequencies ($cm^{-1}$) | $E_{n \rightarrow \pi^*}$ (kcal/mol) | π* Occupancy |
|---|---|---|---|---|
| M06-2X/6-31+G(d,p) | −949.514242 | 25.16 | 2.53 | 0.18602 |
| SMD($H_2O$)-M06-2X/6-31+G(d,p) | −949.538621 | 11.97 | 0.83 | 0.19645 |
| SMD($H_2O$)-M06-2X/def2-TZVP | −949.874046 | 9.99 | 0.76 | 0.19694 |
| SMD($H_2O$)-ωB97XD/6-31+G(d,p) | −949.627242 | 12.26 | 0.72 | 0.20831 |
| M06-2X/def2-TZVP | −949.851189 | 13.81 | 1.0 | 0.18272 |

**Table S2.** Benchmark study on compound **1** with *exo* ring pucker.

| Method | Sum of Electronic and Thermal Free Energies (Hartree) | Lowest Frequencies ($cm^{-1}$) | $E_{n \rightarrow \pi^*}$ (kcal/mol) | π* Occupancy |
|---|---|---|---|---|
| M06-2X/6-31+G(d,p) | −949.513738 | 29.56 | 2.99 | 0.18742 |
| SMD($H_2O$)-M06-2X/6-31+G(d,p) | −949.538300 | 18.54 | 0.90 | 0.19982 |
| SMD($H_2O$)-M06-2X/def2-TZVP | −949.873396 | 17.24 | 0.81 | 0.19950 |

**Table S3.** Benchmark study on compound **2** with *endo* ring pucker.

| Method | Sum of Electronic and Thermal Free Energies (Hartree) | Lowest Frequencies ($cm^{-1}$) | $E_{n\to\pi^*}$ (kcal/mol) | π* Occupancy |
|---|---|---|---|---|
| M06-2X/6-31+G(d,p) | −988.794331 | 23.04 | 2.71 | 0.18416 |
| SMD($H_2O$)-M06-2X/6-31+G(d,p) | −988.818950 | 15.74 | 1.03 | 0.19616 |
| SMD($H_2O$)-M06-2X/def2-TZVP | −989.165610 | 16.17 | 1.02 | 0.19616 |
| SMD($H_2O$)-ωB97XD/6-31+G(d,p) | −988.917872 | 11.1 | 0.78 | 0.20706 |
| M06-2X/def2-TZVP | −989.142939 | 23.66 | 1.99 | 0.18227 |

**Table S4.** Benchmark study on compound **2** with *exo* ring pucker.

| Method | Sum of Electronic and Thermal Free Energies (Hartree) | Lowest Frequencies ($cm^{-1}$) | $E_{n\to\pi^*}$ (kcal/mol) | π* Occupancy |
|---|---|---|---|---|
| M06-2X/6-31+G(d,p) | −988.794040 | 26.72 | 3.66 | 0.18758 |
| SMD($H_2O$)-M06-2X/6-31+G(d,p) | −988.818973 | 13.07 | 2.05 | 0.20371 |
| SMD($H_2O$)-M06-2X/def2-TZVP | −989.165669 | 12.98 | 1.71 | 0.20142 |

**Table S5.** Benchmark study on compound **3** with *endo* ring pucker.

| Method | Sum of Electronic and Thermal Free Energies (Hartree) | Lowest Frequencies ($cm^{-1}$) | $E_{n\rightarrow\pi^*}$ (kcal/mol) | π* Occupancy |
|---|---|---|---|---|
| M06-2X/6-31+G(d,p) | −1106.598562 | 18.59 | 3.39 | 0.18465 |
| SMD($H_2O$)-M06-2X/6-31+G(d,p) | −1106.619519 | 18.31 | 2.32 | 0.19957 |
| SMD($H_2O$)-M06-2X/def2-TZVP | −1107.000335 | 19.83 | 1.82 | 0.19819 |
| SMD($H_2O$)-ωB97XD/6-31+G(d,p) | −1106.746782 | 18.09 | 2.53 | 0.21099 |
| M06-2X/def2-TZVP | −1106.980767 | 19.94 | 2.53 | 0.18253 |

**Table S6.** Benchmark study on compound **3** with *exo* ring pucker.

| Method | Sum of Electronic and Thermal Free Energies (Hartree) | Lowest Frequencies ($cm^{-1}$) | $E_{n\rightarrow\pi^*}$ (kcal/mol) | π* Occupancy |
|---|---|---|---|---|
| M06-2X/6-31+G(d,p) | −1106.59775 | 23.82 | 4.12 | 0.18757 |
| SMD($H_2O$)-M06-2X/6-31+G(d,p) | −1106.61889 | 17.78 | 3.24 | 0.20502 |
| SMD($H_2O$)-M06-2X/def2-TZVP | −1107.000354 | 13.7 | 2.52 | 0.20173 |

**Table S7.** Computational Parameters of Esters **1**−**3** with Geometries Optimized at the M06-2X/6-31+G(d,p) Level of Theory.

| Parameter | **1** $C^{\gamma}$-endo | **1** $C^{\gamma}$-exo | **2** $C^{\gamma}$-endo | **2** $C^{\gamma}$-exo | **3** $C^{\gamma}$-endo | **3** $C^{\gamma}$-exo |
|---|---|---|---|---|---|---|
| $E_{n\rightarrow\pi^*}$ (kcal/mol)[a] | 2.53 | 2.99 | 2.71 | 3.66 | 3.39 | 4.12 |
| **Angles (°)** | | | | | | |
| $\theta$ | 97.13 | 97.90 | 97.99 | 98.29 | 98.88 | 99.65 |
| $\chi_C$ | 2.29 | 1.94 | 2.18 | 2.62 | 2.81 | 3.09 |
| **Distances (Å)** | | | | | | |
| $d$ | 2.724 | 2.708 | 2.699 | 2.641 | 2.628 | 2.585 |
| $\Delta$ | 0.016 | 0.013 | 0.015 | 0.018 | 0.020 | 0.022 |

[a]$E_{n\rightarrow\pi^*}$ values are contributions from *p*-type lone pairs as calculated with second-order perturbation theory.

**Table S8.** Computational Parameters of Esters **1**−**3** with Geometries Optimized at the SMD($H_2O$)-M06-2X/6-31+G(d,p) Level of Theory.

| Parameter | **1** $C^{\gamma}$-endo | **1** $C^{\gamma}$-exo | **2** $C^{\gamma}$-endo | **2** $C^{\gamma}$-exo | **3** $C^{\gamma}$-endo | **3** $C^{\gamma}$-exo |
|---|---|---|---|---|---|---|
| $E_{n\rightarrow\pi^*}$ (kcal/mol)[a] | 0.83 | 0.90 | 1.03 | 2.05 | 2.32 | 3.24 |
| **Angles (°)** | | | | | | |
| $\theta$ | 88.00 | 87.70 | 91.03 | 92.48 | 95.59 | 97.23 |
| $\chi_C$ | 2.38 | 2.42 | 2.61 | 2.78 | 3.42 | 3.69 |
| **Distances (Å)** | | | | | | |
| $d$ | 2.946 | 2.940 | 2.903 | 2.777 | 2.720 | 2.656 |
| $\Delta$ | 0.017 | 0.017 | 0.019 | 0.019 | 0.024 | 0.026 |

[a]$E_{n\rightarrow\pi^*}$ values are contributions from *p*-type lone pairs as calculated with second-order perturbation theory.

**Energies, Frequencies, and Coordinates of Computed Structures.** The optimized structures reported herein are available in the ioChem-BD repository.[20] See: https://doi.org/10.19061/iochem-bd-6-370.

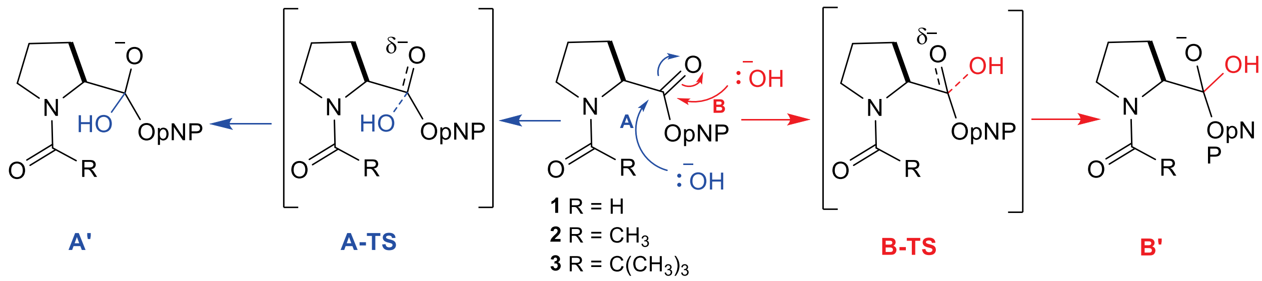


| | A' | A-TS | | B-TS | B' |
|---|---|---|---|---|---|
| **1:** | -7.2 | 11.2 | 0.0 | 12.1 | -7.9 |
| **2:** | -5.8 | 14.7 | 0.0 | 12.6 | -5.0 |
| **3:** | -3.1 | 14.6 | 0.0 | 13.1 | -3.0 |

**Figure S9.** Computed (SMD($H_2O$)-M06-2x/6-31+G(d,p)) relative free energies (kcal/mol) for minima and TSSs involved in the nucleophilic attack on the cis isomers (with C$^{\gamma}$-endo ring pucker) of *N*-acylated (2*S*)-proline *p*-nitrophenyl esters **1**–**3** from the front (**A**) and the back (**B**).

**Table S9.** Key Bond Lengths and Angles in Computational and Experimental Structures of Esters **1**–**3**.

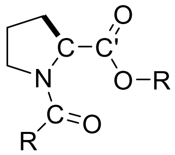


| Parameter | Computational[a] | | | Experimental[b] | |
|---|---|---|---|---|---|
| | **1** C$^{\gamma}$-endo | **2** C$^{\gamma}$-exo | **3** C$^{\gamma}$-endo | **2** C$^{\gamma}$-exo | **3** C$^{\gamma}$-endo |
| **Bond Lengths (Å)** | | | | | |
| C′=O | 1.200 | 1.200 | 1.201 | 1.194 | 1.192 |
| C=O | 1.222 | 1.228 | 1.231 | 1.232 | 1.235 |
| Amide C–N | 1.352 | 1.359 | 1.368 | 1.345 | 1.353 |
| Ring N–C | 1.458 | 1.460 | 1.461 | 1.458 | 1.467 |
| C–C′ | 1.524 | 1.522 | 1.522 | 1.512 | 1.518 |
| **Bond Angles (°)** | | | | | |
| R–C=O | 122.93 | 122.73 | 120.55 | 122.95 | 120.53 |
| N–C=O | 123.03 | 120.49 | 118.20 | 121.15 | 118.47 |
| N–C–R | 114.04 | 116.78 | 121.24 | 115.90 | 120.97 |
| C–C′=O | 124.86 | 124.50 | 125.26 | 127.37 | 127.18 |
| O–C′=O | 124.32 | 124.44 | 124.03 | 124.21 | 123.58 |
| C–C′–O | 110.77 | 111.01 | 110.65 | 108.24 | 109.16 |

[a]Geometries were optimized at the M06-2X/6-31+G(d,p) level of theory.
[b]Values were determined by X-ray diffraction analysis (Figure 5).

**Computational NMR Data for Esters 1–3**

**Table S10.** Comparison of the calculated and experimental chemical shifts ($\delta$, ppm) of each numbered nucleus ($^{13}$C and $^{1}$H) in ester **1** (SMD($CHCl_3$)-mPW1PW91/6-311+G(2d,p)//SMD($H_2O$)-M06-2X/6-31+G(d,p)). Computed chemical shifts ($\delta_{calc}$) were weighted using a Boltzmann-weighted average. One of the conformers of ester **1** is depicted below as a representative structure to show the nucleus numbers. Due to the overlap of two sets of chemical shifts from cis and trans isomer, the assignment comparison was not conducted for protons in the 1–5 ppm chemical shift region.

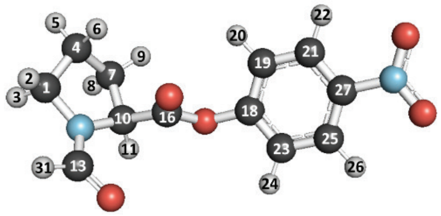


| C# | $\delta_{calc}$ | $\delta_{exp}$ | Abs. Dev. | H# | $\delta_{calc}$ | $\delta_{exp}$ | Abs. Dev. |
|---|---|---|---|---|---|---|---|
| **1** | 47.41 | 46.51 | 0.9 | **20, 24** | 7.20 | 7.30 | 0.1 |
| **4** | 25.39 | 24.47 | 0.9 | **22, 26** | 8.36 | 8.31 | 0.0 |
| **7** | 30.93 | 29.56 | 1.4 | | | **MAD** | **0.1** |
| **10** | 57.59 | 56.94 | 0.6 | | | | |
| **13** | 161.35 | 161.12 | 0.2 | | | | |
| **16** | 171.71 | 169.64 | 2.1 | | | | |
| **18** | 156.86 | 155.38 | 1.5 | | | | |
| **19, 23** | 122.83 | 122.52 | 0.3 | | | | |
| **21, 25** | 126.10 | 125.38 | 0.7 | | | | |
| **27** | 143.19 | 145.68 | 2.5 | | | | |
| | | **MAD** | **1.1** | | | | |

**Table S11.** Comparison of the calculated and experimental chemical shifts ($\delta$, ppm) of each numbered nucleus ($^{13}C$ and $^{1}H$) in ester **2** (SMD($CHCl_3$)-mPW1PW91/6-311+G(2d,p)//SMD($H_2O$)-M06-2X/6-31+G(d,p)). Computed chemical shifts ($\delta_{calc}$) were weighted using a Boltzmann-weighted average. One of the conformers of ester **2** is depicted below as a representative structure to show the nucleus numbers. Due to the overlap of two sets of chemical shifts from cis and trans isomer, the assignment comparison was not conducted for some protons in the 1–5 ppm chemical shift region.

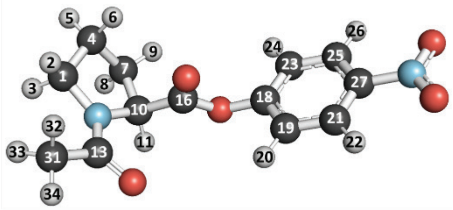


| C# | $\delta_{calc}$ | $\delta_{exp}$ | Abs. Dev. | H# | $\delta_{calc}$ | $\delta_{exp}$ | Abs. Dev. |
|---|---|---|---|---|---|---|---|
| **1** | 48.53 | 47.99 | 0.5 | **20, 24** | 7.21 | 7.29 | 0.1 |
| **4** | 26.52 | 25.29 | 1.2 | **22, 26** | 8.35 | 8.31 | 0.0 |
| **7** | 31.15 | 31.80 | 0.7 | **32–34** | 2.02 | 2.03 | 0.0 |
| **10** | 59.26 | 59.03 | 0.2 | | | **MAD** | **0.0** |
| **13** | 169.78 | 170.07 | 0.3 | | | | |
| **16** | 172.84 | 170.41 | 2.4 | | | | |
| **18** | 157.09 | 155.61 | 1.5 | | | | |
| **19, 23** | 122.97 | 122.57 | 0.4 | | | | |
| **21, 25** | 126.09 | 125.58 | 0.5 | | | | |
| **27** | 143.20 | 145.60 | 2.4 | | | | |
| **31** | 21.87 | 22.25 | 0.4 | | | | |
| | | **MAD** | **1.0** | | | | |

**Table S12.** Comparison of the calculated and experimental chemical shifts ($\delta$, ppm) of each numbered nucleus ($^{13}C$ and $^{1}H$) in ester **3** (SMD($CHCl_3$)-mPW1PW91/6-311+G(2d,p)//M06-2X/6-31+G(d,p)). Computed chemical shifts ($\delta_{calc}$) were weighted using a Boltzmann-weighted average. One of the conformers of ester **3** is depicted below as a representative structure to show the nucleus numbers.

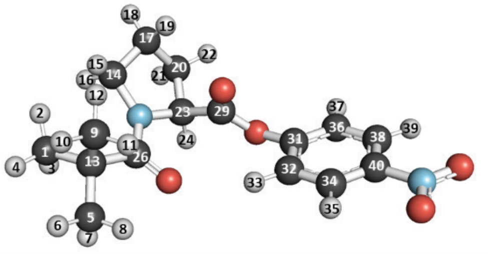


| C# | $\delta_{calc}$ | $\delta_{exp}$ | Abs. Dev. | H# | $\delta_{calc}$ | $\delta_{exp}$ | Abs. Dev. |
|---|---|---|---|---|---|---|---|
| **1, 5, 9** | 24.79 | 27.31 | 2.5 | **2–4, 6–8, 10–12** | 1.22 | 1.29 | 0.1 |
| **13** | 40.27 | 38.86 | 1.4 | | | | |
| **14** | 48.88 | 48.48 | 0.4 | **15, 16** | 3.68 | 3.82 | 0.1 |
| **17** | 27.42 | 26.47 | 1.0 | **18, 19** | 2.09 | 2.06 | 0.0 |
| **20** | 28.43 | 27.86 | 0.6 | **21** | 2.11 | 2.31 | 0.2 |
| **23** | 62.56 | 61.42 | 1.1 | **22** | 2.04 | 2.19 | 0.2 |
| **26** | 176.07 | 177.46 | 1.4 | **24** | 4.37 | 4.67 | 0.3 |
| **29** | 173.48 | 170.84 | 2.6 | **33, 37** | 7.25 | 7.32 | 0.1 |
| **31** | 157.81 | 155.83 | 2.0 | **35, 39** | 8.30 | 8.25 | 0.0 |
| **32, 36** | 121.93 | 122.55 | 0.6 | | | **MAD** | **0.1** |
| **34, 38** | 125.49 | 125.31 | 0.2 | | | | |
| **40** | 144.03 | 145.48 | 1.5 | | | | |
| | | **MAD** | **1.3** | | | | |

**Table S13.** Comparison of the calculated and experimental chemical shifts ($\delta$, ppm) of each numbered nucleus ($^{13}C$ and $^{1}H$) in ester **3** (SMD($CHCl_3$)-mPW1PW91/6-311+G(2d,p)// SMD($H_2O$)-M06-2X/6-31+G(d,p)). Computed chemical shifts ($\delta_{calc}$) were weighted using a Boltzmann-weighted average. One of the conformers of ester **3** is depicted below as a representative structure to show the nucleus numbers.

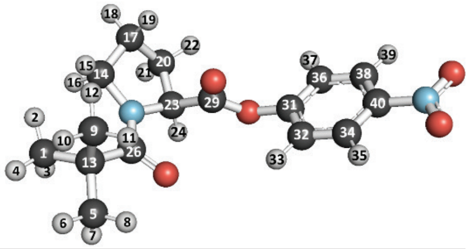


| C# | $\delta_{calc}$ | $\delta_{exp}$ | Abs. Dev. | H# | $\delta_{calc}$ | $\delta_{exp}$ | Abs. Dev. |
|---|---|---|---|---|---|---|---|
| **1, 5, 9** | 25.17 | 27.31 | 2.1 | **2–4, 6–8, 10–12** | 1.24 | 1.29 | 0.1 |
| **13** | 40.19 | 38.86 | 1.3 | | | | |
| **14** | 49.77 | 48.48 | 1.3 | **15, 16** | 3.69 | 3.82 | 0.1 |
| **17** | 27.39 | 26.47 | 0.9 | **18, 19** | 1.99 | 2.06 | 0.1 |
| **20** | 28.99 | 27.86 | 1.1 | **21** | 2.19 | 2.31 | 0.1 |
| **23** | 62.00 | 61.42 | 0.6 | **22** | 1.99 | 2.19 | 0.2 |
| **26** | 176.98 | 177.46 | 0.5 | **24** | 4.39 | 4.67 | 0.3 |
| **29** | 172.99 | 170.84 | 2.2 | **33, 37** | 7.21 | 7.32 | 0.1 |
| **31** | 157.22 | 155.83 | 1.4 | **35, 39** | 8.35 | 8.25 | 0.1 |
| **32, 36** | 122.76 | 122.55 | 0.2 | | | **MAD** | **0.1** |
| **34, 38** | 125.96 | 125.31 | 0.6 | | | | |
| **40** | 142.96 | 145.48 | 2.5 | | | | |
| | | **MAD** | **1.2** | | | | |

**X-Ray Crystal Structures**

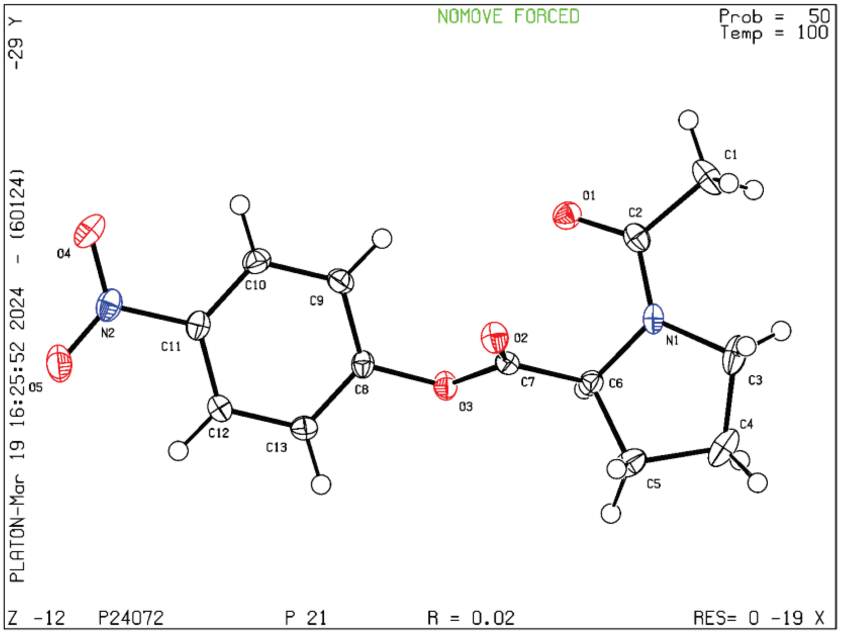


**Figure S10.** Oak Ridge thermal ellipsoid plot (ORTEP) of *N*-acetyl-(2*S*)-proline *p*-nitrophenyl ester (**2**) from X-ray crystallography.

**Table S14.** Crystal data and structure refinement for *N*-acetyl-(2*S*)-proline *p*-nitrophenyl ester (**2**).

| | |
|---|---|
| CCDC number | 2350603 |
| Identification code | P24072 |
| Empirical formula | $C_{13}H_{14}N_2O_5$ |
| Formula weight | 278.26 |
| Temperature | 100(2) K |
| Wavelength | 1.54178 Å |
| Crystal system | Monoclinic |
| Space group | $P2_1$ |
| Unit cell dimensions | |
| $a$ = 7.7315(2) Å | $\alpha$ = 90° |
| $b$ = 11.1977(3) Å | $\beta$ = 115.9420(9)° |
| $c$ = 8.2434(3) Å | $\gamma$ = 90° |
| Volume | 641.76(3) $Å^3$ |
| Z | 2 |
| Density (calculated) | 1.440 $Mg/m^3$ |
| Absorption coefficient | 0.949 $mm^{-1}$ |
| $F_{000}$ | 292 |
| Crystal size | 0.255 × 0.250 × 0.230 $mm^3$ |
| Theta range for data collection | 5.969 to 77.459° |
| Index ranges | $-9 \le h \le 9$, $-14 \le k \le 14$, $-10 \le l \le 10$ |
| Reflections collected | 38344 |
| Independent reflections | 2711 [$R_{int}$ = 0.0222] |
| Completeness to theta = 67.679° | 99.8% |
| Absorption correction | Semi-empirical from equivalents |
| Refinement method | Full-matrix least-squares on $F^2$ |
| Data / restraints / parameters | 2711/1/183 |
| Goodness-of-fit on $F^2$ | 1.052 |
| Final $R$ indices [$I > 2\sigma_I$] | $R1$ = 0.0210, w$R2$ = 0.0554 |
| $R$ indices (all data) | $R1$ = 0.0210, w$R2$ = 0.0554 |
| Absolute structure parameter | 0.050(17) |
| Extinction coefficient | 0.0099(11) |
| Largest diff. peak and hole | 0.214 and −0.121 e·$Å^{-3}$ |

**Table S15.** Atomic coordinates (×$10^4$) and equivalent isotropic displacement parameters (Å$^2$ × $10^3$) for ester **2**. $U_{eq}$ is defined as one-third of the trace of the orthogonalized $U^{ij}$ tensor.

| | *x* | *y* | *z* | $U_{eq}$ |
|---|---|---|---|---|
| O(1) | 9710(1) | 6256(1) | 7017(1) | 20(1) |
| O(2) | 7677(1) | 5170(1) | 9168(1) | 21(1) |
| O(3) | 6694(1) | 4021(1) | 6650(1) | 19(1) |
| O(4) | −1345(2) | 6541(1) | 3008(1) | 26(1) |
| O(5) | −2203(2) | 4736(1) | 3274(2) | 29(1) |
| N(1) | 11482(2) | 4977(1) | 9213(2) | 18(1) |
| N(2) | −980(2) | 5493(1) | 3474(2) | 21(1) |
| C(1) | 12916(2) | 6873(2) | 9074(2) | 30(1) |
| C(2) | 11230(2) | 6024(1) | 8340(2) | 19(1) |
| C(3) | 13254(2) | 4545(2) | 10730(2) | 27(1) |
| C(4) | 12913(2) | 3209(2) | 10728(2) | 29(1) |
| C(5) | 10732(2) | 3101(1) | 10011(2) | 24(1) |
| C(6) | 10002(2) | 4056(1) | 8524(2) | 16(1) |
| C(7) | 8048(2) | 4530(1) | 8204(2) | 16(1) |
| C(8) | 4804(2) | 4426(1) | 5966(2) | 16(1) |
| C(9) | 4379(2) | 5624(1) | 5520(2) | 19(1) |
| C(10) | 2469(2) | 5982(1) | 4698(2) | 19(1) |
| C(11) | 1049(2) | 5124(1) | 4335(2) | 18(1) |
| C(12) | 1465(2) | 3932(1) | 4769(2) | 18(1) |
| C(13) | 3383(2) | 3579(1) | 5608(2) | 18(1) |

**Table S16.** Bond lengths [Å] and angles [°] for ester **2**.

| | | | |
|---|---|---|---|
| O(1)-C(2) | 1.2314(18) | C(12)-H(12) | 0.9500 |
| O(2)-C(7) | 1.1945(18) | C(13)-H(13) | 0.9500 |
| O(3)-C(7) | 1.3737(16) | | |
| O(3)-C(8) | 1.3919(15) | C(7)-O(3)-C(8) | 118.65(10) |
| O(4)-N(2) | 1.2282(17) | C(2)-N(1)-C(6) | 120.17(11) |
| O(5)-N(2) | 1.2261(17) | C(2)-N(1)-C(3) | 127.08(13) |
| N(1)-C(2) | 1.3449(19) | C(6)-N(1)-C(3) | 112.23(12) |
| N(1)-C(6) | 1.4580(18) | O(5)-N(2)-O(4) | 124.06(13) |
| N(1)-C(3) | 1.4750(17) | O(5)-N(2)-C(11) | 117.87(13) |
| N(2)-C(11) | 1.4699(17) | O(4)-N(2)-C(11) | 118.07(13) |
| C(1)-C(2) | 1.510(2) | C(2)-C(1)-H(1A) | 109.5 |
| C(1)-H(1A) | 0.9800 | C(2)-C(1)-H(1B) | 109.5 |
| C(1)-H(1B) | 0.9800 | H(1A)-C(1)-H(1B) | 109.5 |
| C(1)-H(1C) | 0.9800 | C(2)-C(1)-H(1C) | 109.5 |
| C(3)-C(4) | 1.519(3) | H(1A)-C(1)-H(1C) | 109.5 |
| C(3)-H(3A) | 0.9900 | H(1B)-C(1)-H(1C) | 109.5 |
| C(3)-H(3B) | 0.9900 | O(1)-C(2)-N(1) | 121.11(12) |
| C(4)-C(5) | 1.528(2) | O(1)-C(2)-C(1) | 122.98(14) |
| C(4)-H(4A) | 0.9900 | N(1)-C(2)-C(1) | 115.91(13) |
| C(4)-H(4B) | 0.9900 | N(1)-C(3)-C(4) | 103.03(13) |
| C(5)-C(6) | 1.5363(19) | N(1)-C(3)-H(3A) | 111.2 |
| C(5)-H(5A) | 0.9900 | C(4)-C(3)-H(3A) | 111.2 |
| C(5)-H(5B) | 0.9900 | N(1)-C(3)-H(3B) | 111.2 |
| C(6)-C(7) | 1.5118(18) | C(4)-C(3)-H(3B) | 111.2 |
| C(6)-H(6) | 1.0000 | H(3A)-C(3)-H(3B) | 109.1 |
| C(8)-C(13) | 1.3824(19) | C(3)-C(4)-C(5) | 103.93(12) |
| C(8)-C(9) | 1.391(2) | C(3)-C(4)-H(4A) | 111.0 |
| C(9)-C(10) | 1.388(2) | C(5)-C(4)-H(4A) | 111.0 |
| C(9)-H(9) | 0.9500 | C(3)-C(4)-H(4B) | 111.0 |
| C(10)-C(11) | 1.3899(19) | C(5)-C(4)-H(4B) | 111.0 |
| C(10)-H(10) | 0.9500 | H(4A)-C(4)-H(4B) | 109.0 |
| C(11)-C(12) | 1.383(2) | C(4)-C(5)-C(6) | 102.22(12) |
| C(12)-C(13) | 1.3911(19) | C(4)-C(5)-H(5A) | 111.3 |
| C(6)-C(5)-H(5A) | 111.3 | | |
| C(4)-C(5)-H(5B) | 111.3 | | |
| C(6)-C(5)-H(5B) | 111.3 | | |
| H(5A)-C(5)-H(5B) | 109.2 | | |

| | |
|---|---|
| N(1)-C(6)-C(7) | 111.96(11) |
| N(1)-C(6)-C(5) | 103.26(11) |
| C(7)-C(6)-C(5) | 111.95(11) |
| N(1)-C(6)-H(6) | 109.8 |
| C(7)-C(6)-H(6) | 109.8 |
| C(5)-C(6)-H(6) | 109.8 |
| O(2)-C(7)-O(3) | 124.22(12) |
| O(2)-C(7)-C(6) | 127.38(12) |
| O(3)-C(7)-C(6) | 108.22(11) |
| C(13)-C(8)-C(9) | 122.05(13) |
| C(13)-C(8)-O(3) | 117.31(12) |
| C(9)-C(8)-O(3) | 120.41(12) |
| C(10)-C(9)-C(8) | 119.10(13) |
| C(10)-C(9)-H(9) | 120.5 |
| C(8)-C(9)-H(9) | 120.4 |
| C(9)-C(10)-C(11) | 118.46(13) |
| C(9)-C(10)-H(10) | 120.8 |
| C(11)-C(10)-H(10) | 120.8 |
| C(12)-C(11)-C(10) | 122.64(13) |
| C(12)-C(11)-N(2) | 118.35(12) |
| C(10)-C(11)-N(2) | 119.01(13) |
| C(11)-C(12)-C(13) | 118.64(13) |
| C(11)-C(12)-H(12) | 120.7 |
| C(13)-C(12)-H(12) | 120.7 |
| C(8)-C(13)-C(12) | 119.11(13) |
| C(8)-C(13)-H(13) | 120.4 |
| C(12)-C(13)-H(13) | 120.4 |

**Table S17.** Anisotropic displacement parameters (Å$^2$ × 10$^3$) for ester **2**. The anisotropic displacement factor exponent takes the form: $-2\pi^2[h^2 a^{*2}U^{11} + ... + 2 h k a^* b^* U^{12}]$.

| | $U^{11}$ | $U^{22}$ | $U^{33}$ | $U^{23}$ | $U^{13}$ | $U^{12}$ |
|---|---|---|---|---|---|---|
| O(1) | 22(1) | 20(1) | 18(1) | 1(1) | 7(1) | 1(1) |
| O(2) | 19(1) | 24(1) | 21(1) | −4(1) | 10(1) | 0(1) |
| O(3) | 14(1) | 19(1) | 22(1) | −5(1) | 6(1) | 0(1) |
| O(4) | 26(1) | 27(1) | 21(1) | 3(1) | 7(1) | 11(1) |
| O(5) | 16(1) | 34(1) | 32(1) | −4(1) | 6(1) | −1(1) |
| N(1) | 11(1) | 23(1) | 18(1) | −1(1) | 4(1) | 0(1) |
| N(2) | 18(1) | 26(1) | 16(1) | −2(1) | 5(1) | 4(1) |
| C(1) | 24(1) | 32(1) | 36(1) | −9(1) | 16(1) | −12(1) |
| C(2) | 18(1) | 21(1) | 20(1) | −4(1) | 11(1) | −3(1) |
| C(3) | 17(1) | 38(1) | 19(1) | −3(1) | 1(1) | 7(1) |
| C(4) | 27(1) | 36(1) | 21(1) | 5(1) | 7(1) | 16(1) |
| C(5) | 28(1) | 23(1) | 23(1) | 6(1) | 14(1) | 7(1) |
| C(6) | 16(1) | 17(1) | 17(1) | 1(1) | 8(1) | 2(1) |
| C(7) | 15(1) | 15(1) | 18(1) | 1(1) | 8(1) | −1(1) |
| C(8) | 14(1) | 18(1) | 16(1) | −2(1) | 6(1) | 1(1) |
| C(9) | 18(1) | 16(1) | 22(1) | −1(1) | 9(1) | −3(1) |
| C(10) | 21(1) | 15(1) | 20(1) | 0(1) | 7(1) | 2(1) |
| C(11) | 16(1) | 22(1) | 15(1) | −1(1) | 6(1) | 2(1) |
| C(12) | 16(1) | 19(1) | 18(1) | −2(1) | 7(1) | −2(1) |
| C(13) | 19(1) | 14(1) | 20(1) | −1(1) | 9(1) | 0(1) |

**Table S18.** Hydrogen coordinates (×10[4]) and isotropic displacement parameters ($Å^2 \times 10^3$) for ester **2**.

| | $x$ | $y$ | $z$ | $U_{eq}$ |
|---|---|---|---|---|
| H(1A) | 12555 | 7627 | 8405 | 45 |
| H(1B) | 14005 | 6518 | 8939 | 45 |
| H(1C) | 13284 | 7024 | 10354 | 45 |
| H(3A) | 13425 | 4913 | 11882 | 33 |
| H(3B) | 14400 | 4718 | 10527 | 33 |
| H(4A) | 13579 | 2876 | 11962 | 35 |
| H(4B) | 13368 | 2788 | 9932 | 35 |
| H(5A) | 10317 | 3274 | 10965 | 29 |
| H(5B) | 10279 | 2295 | 9512 | 29 |
| H(6) | 9943 | 3718 | 7378 | 20 |
| H(9) | 5382 | 6188 | 5775 | 22 |
| H(10) | 2140 | 6796 | 4391 | 23 |
| H(12) | 462 | 3365 | 4499 | 21 |
| H(13) | 3711 | 2768 | 5932 | 22 |

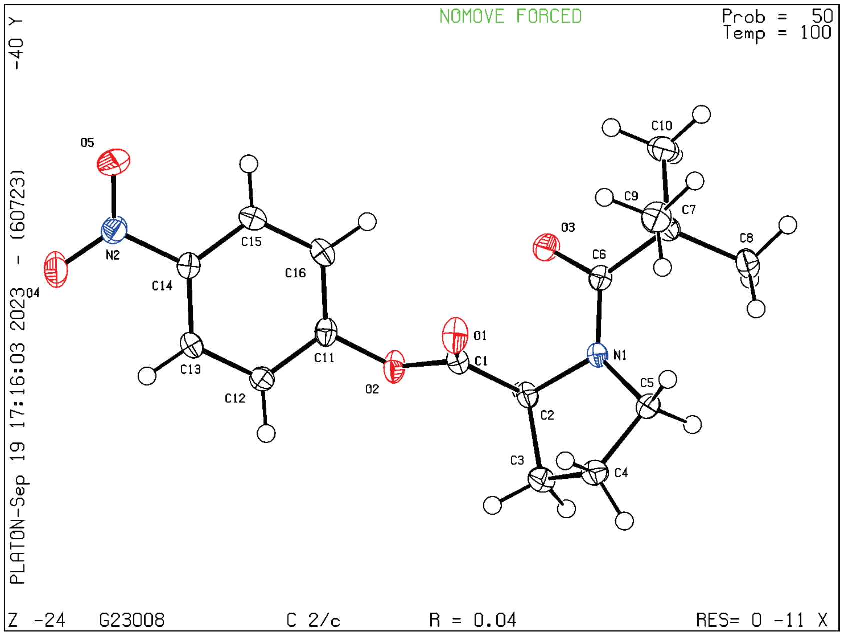


**Figure S11.** Oak Ridge thermal ellipsoid plot (ORTEP) of *N*-pivaloyl-(2*S*)-proline *p*-nitrophenyl ester (**3**) from X-ray crystallography.

**Table S19.** Crystal data and structure refinement for *N*-pivaloyl-(2*S*)-proline *p*-nitrophenyl ester (**3**).

| | |
|---|---|
| CCDC number | 2350600 |
| Identification code | G23008 |
| Empirical formula | $C_{16}H_{20}N_2O_5$ |
| Formula weight | 320.34 |
| Temperature | 100(2) K |
| Wavelength | 1.54178 Å |
| Crystal system | Monoclinic |
| Space group | *C*2/*c* |
| Unit cell dimensions | |
| $a$ = 21.7908(7) Å | $\alpha$ = 90° |
| $b$ = 7.4882(2) Å | $\beta$ = 111.8599(12)° |
| $c$ = 21.0673(7) Å | $\gamma$ = 90° |
| Volume | 3190.46(17) $Å^3$ |
| $Z$ | 8 |
| Density (calculated) | 1.334 $Mg/m^3$ |
| Absorption coefficient | 0.832 $mm^{-1}$ |
| $F_{000}$ | 1360 |
| Crystal size | 0.365 × 0.330 × 0.135 $mm^3$ |
| Theta range for data collection | 4.372 to 74.482° |
| Index ranges | $-27 \leq h \leq 26$, $-9 \leq k \leq 9$, $-26 \leq l \leq 25$ |
| Reflections collected | 23431 |
| Independent reflections | 3266 [$R_{int}$ = 0.0393] |
| Completeness to theta = 67.679° | 100.0% |
| Absorption correction | Semi-empirical from equivalents |
| Refinement method | Full-matrix least-squares on $F^2$ |
| Data/restraints/parameters | 3266/0/211 |
| Goodness-of-fit on $F^2$ | 1.070 |
| Final $R$ indices [$I > 2\sigma_I$] | $R1$ = 0.0373, w$R2$ = 0.0951 |
| $R$ indices (all data) | $R1$ = 0.0388, w$R2$ = 0.0964 |
| Extinction coefficient | n/a |
| Largest diff. peak and hole | 0.294 and −0.312 e·$Å^{-3}$ |

**Table S20.** Atomic coordinates (×10$^4$) and equivalent isotropic displacement parameters (Å$^2$ × 10$^3$) for ester **3**. $U_{eq}$ is defined as one-third of the trace of the orthogonalized $U^{ij}$ tensor.

| | *x* | *y* | *z* | $U_{eq}$ |
|---|---|---|---|---|
| O(1)6690(1) | 2326(1) | 3512(1) | 26(1) | |
| O(2)6449(1) | 1681(1) | 2396(1) | 24(1) | |
| O(3)7980(1) | 3208(1) | 3274(1) | 25(1) | |
| O(4)3934(1) | 6350(1) | 1004(1) | 35(1) | |
| O(5)4600(1) | 8388(1) | 1595(1) | 44(1) | |
| N(1)7936(1) | 672(1) | 3805(1) | 17(1) | |
| N(2)4474(1) | 6836(1) | 1407(1) | 23(1) | |
| C(1)6816(1) | 1540(2) | 3083(1) | 19(1) | |
| C(2)7364(1) | 183(2) | 3193(1) | 18(1) | |
| C(3)7170(1) | −1674(2) | 3365(1) | 23(1) | |
| C(4)7402(1) | −1655(2) | 4143(1) | 24(1) | |
| C(5)8052(1) | −635(2) | 4362(1) | 21(1) | |
| C(6)8208(1) | 2292(2) | 3797(1) | 18(1) | |
| C(7)8773(1) | 3013(2) | 4435(1) | 20(1) | |
| C(8)9332(1) | 1654(2) | 4755(1) | 24(1) | |
| C(9)8474(1) | 3626(2) | 4954(1) | 28(1) | |
| C(10) | 9066(1) | 4652(2) | 4210(1) | 31(1) |
| C(11) | 5957(1) | 2983(2) | 2188(1) | 19(1) |
| C(12) | 5310(1) | 2430(2) | 1852(1) | 19(1) |
| C(13) | 4817(1) | 3706(2) | 1594(1) | 18(1) |
| C(14) | 4994(1) | 5487(2) | 1687(1) | 18(1) |
| C(15) | 5640(1) | 6053(2) | 2031(1) | 21(1) |
| C(16) | 6129(1) | 4772(2) | 2283(1) | 21(1) |

**Table S21.** Bond lengths [Å] and angles [°] for ester **3**.

| | | | |
|---|---|---|---|
| O(1)-C(1) | 1.1916(14) | C(10)-H(10C) | 0.9800 |
| O(2)-C(1) | 1.3734(13) | C(11)-C(12) | 1.3845(15) |
| O(2)-C(11) | 1.3938(13) | C(11)-C(16) | 1.3853(16) |
| O(3)-C(6) | 1.2349(13) | C(12)-C(13) | 1.3876(15) |
| O(4)-N(2) | 1.2227(13) | C(12)-H(12) | 0.9500 |
| O(5)-N(2) | 1.2249(14) | C(13)-C(14) | 1.3816(16) |
| N(1)-C(6) | 1.3526(14) | C(13)-H(13) | 0.9500 |
| N(1)-C(2) | 1.4674(13) | C(14)-C(15) | 1.3895(15) |
| N(1)-C(5) | 1.4767(14) | C(15)-C(16) | 1.3845(16) |
| N(2)-C(14) | 1.4666(14) | C(15)-H(15) | 0.9500 |
| C(1)-C(2) | 1.5181(15) | C(16)-H(16) | 0.9500 |
| C(2)-C(3) | 1.5349(15) | | |
| C(2)-H(2) | 1.0000 | C(1)-O(2)-C(11) | 117.58(8) |
| C(3)-C(4) | 1.5262(16) | C(6)-N(1)-C(2) | 116.50(9) |
| C(3)-H(3A) | 0.9900 | C(6)-N(1)-C(5) | 131.13(9) |
| C(3)-H(3B) | 0.9900 | C(2)-N(1)-C(5) | 111.76(9) |
| C(4)-C(5) | 1.5226(15) | O(4)-N(2)-O(5) | 123.26(10) |
| C(4)-H(4A) | 0.9900 | O(4)-N(2)-C(14) | 118.23(10) |
| C(4)-H(4B) | 0.9900 | O(5)-N(2)-C(14) | 118.50(10) |
| C(5)-H(5A) | 0.9900 | O(1)-C(1)-O(2) | 123.59(10) |
| C(5)-H(5B) | 0.9900 | O(1)-C(1)-C(2) | 127.18(10) |
| C(6)-C(7) | 1.5431(15) | O(2)-C(1)-C(2) | 109.15(9) |
| C(7)-C(8) | 1.5353(16) | N(1)-C(2)-C(1) | 109.30(9) |
| C(7)-C(10) | 1.5361(16) | N(1)-C(2)-C(3) | 103.76(8) |
| C(7)-C(9) | 1.5388(16) | C(1)-C(2)-C(3) | 111.92(9) |
| C(8)-H(8A) | 0.9800 | N(1)-C(2)-H(2) | 110.6 |
| C(8)-H(8B) | 0.9800 | C(1)-C(2)-H(2) | 110.6 |
| C(8)-H(8C) | 0.9800 | C(3)-C(2)-H(2) | 110.6 |
| C(9)-H(9A) | 0.9800 | C(4)-C(3)-C(2) | 103.55(9) |
| C(9)-H(9B) | 0.9800 | C(4)-C(3)-H(3A) | 111.1 |
| C(9)-H(9C) | 0.9800 | C(2)-C(3)-H(3A) | 111.1 |
| C(10)-H(10A) | 0.9800 | C(4)-C(3)-H(3B) | 111.1 |
| C(10)-H(10B) | 0.9800 | C(2)-C(3)-H(3B) | 111.1 |
| H(3A)-C(3)-H(3B) | 109.0 | C(7)-C(10)-H(10A) | 109.5 |
| C(5)-C(4)-C(3) | 103.20(9) | C(7)-C(10)-H(10B) | 109.5 |
| C(5)-C(4)-H(4A) | 111.1 | H(10A)-C(10)-H(10B) | 109.5 |
| C(3)-C(4)-H(4A) | 111.1 | C(7)-C(10)-H(10C) | 109.5 |
| C(5)-C(4)-H(4B) | 111.1 | H(10A)-C(10)-H(10C) | 109.5 |

| | | | |
|---|---|---|---|
| C(3)-C(4)-H(4B) | 111.1 | H(10B)-C(10)-H(10C) | 109.5 |
| H(4A)-C(4)-H(4B) | 109.1 | C(12)-C(11)-C(16) | 122.18(10) |
| N(1)-C(5)-C(4) | 103.14(9) | C(12)-C(11)-O(2) | 118.00(10) |
| N(1)-C(5)-H(5A) | 111.1 | C(16)-C(11)-O(2) | 119.70(10) |
| C(4)-C(5)-H(5A) | 111.1 | C(11)-C(12)-C(13) | 119.12(10) |
| N(1)-C(5)-H(5B) | 111.1 | C(11)-C(12)-H(12) | 120.4 |
| C(4)-C(5)-H(5B) | 111.1 | C(13)-C(12)-H(12) | 120.4 |
| H(5A)-C(5)-H(5B) | 109.1 | C(14)-C(13)-C(12) | 118.36(10) |
| O(3)-C(6)-N(1) | 118.46(10) | C(14)-C(13)-H(13) | 120.8 |
| O(3)-C(6)-C(7) | 120.53(10) | C(12)-C(13)-H(13) | 120.8 |
| N(1)-C(6)-C(7) | 120.97(9) | C(13)-C(14)-C(15) | 122.90(10) |
| C(8)-C(7)-C(10) | 108.26(10) | C(13)-C(14)-N(2) | 118.41(9) |
| C(8)-C(7)-C(9) | 111.20(9) | C(15)-C(14)-N(2) | 118.69(10) |
| C(10)-C(7)-C(9) | 108.35(10) | C(16)-C(15)-C(14) | 118.35(10) |
| C(8)-C(7)-C(6) | 113.33(9) | C(16)-C(15)-H(15) | 120.8 |
| C(10)-C(7)-C(6) | 107.46(9) | C(14)-C(15)-H(15) | 120.8 |
| C(9)-C(7)-C(6) | 108.07(9) | C(15)-C(16)-C(11) | 119.08(10) |
| C(7)-C(8)-H(8A) | 109.5 | C(15)-C(16)-H(16) | 120.5 |
| C(7)-C(8)-H(8B) | 109.5 | C(11)-C(16)-H(16) | 120.5 |
| H(8A)-C(8)-H(8B) | 109.5 | | |
| C(7)-C(8)-H(8C) | 109.5 | | |
| H(8A)-C(8)-H(8C) | 109.5 | | |
| H(8B)-C(8)-H(8C) | 109.5 | | |
| C(7)-C(9)-H(9A) | 109.5 | | |
| C(7)-C(9)-H(9B) | 109.5 | | |
| H(9A)-C(9)-H(9B) | 109.5 | | |
| C(7)-C(9)-H(9C) | 109.5 | | |
| H(9A)-C(9)-H(9C) | 109.5 | | |
| H(9B)-C(9)-H(9C) | 109.5 | | |

**Table S22.** Anisotropic displacement parameters (Å$^2$ × 10$^3$) for ester **3**. The anisotropic displacement factor exponent takes the form: $-2\pi^2[h^2\, a^{*2} U^{11} + ... + 2\, h\, k\, a^*\, b^*\, U^{12}]$.

| | $U^{11}$ | $U^{22}$ | $U^{33}$ | $U^{23}$ | $U^{13}$ | $U^{12}$ |
|---|---|---|---|---|---|---|
| O(1)24(1) | 36(1) | 19(1) | −1(1) | 8(1) | 9(1) | |
| O(2)21(1) | 28(1) | 16(1) | −4(1) | 1(1) | 8(1) | |
| O(3)29(1) | 24(1) | 17(1) | 5(1) | 4(1) | −1(1) | |
| O(4)24(1) | 35(1) | 33(1) | −6(1) | −4(1) | 9(1) | |
| O(5)35(1) | 18(1) | 67(1) | −1(1) | 6(1) | 2(1) | |
| N(1)15(1) | 19(1) | 14(1) | 1(1) | 2(1) | 0(1) | |
| N(2)23(1) | 22(1) | 24(1) | 1(1) | 7(1) | 3(1) | |
| C(1)15(1) | 23(1) | 18(1) | 0(1) | 4(1) | −1(1) | |
| C(2)15(1) | 21(1) | 16(1) | −2(1) | 3(1) | 1(1) | |
| C(3)22(1) | 21(1) | 24(1) | −3(1) | 6(1) | −2(1) | |
| C(4)25(1) | 21(1) | 24(1) | 2(1) | 6(1) | −2(1) | |
| C(5)22(1) | 19(1) | 20(1) | 4(1) | 4(1) | 0(1) | |
| C(6)18(1) | 19(1) | 16(1) | 0(1) | 6(1) | 2(1) | |
| C(7)21(1) | 19(1) | 18(1) | −1(1) | 4(1) | −2(1) | |
| C(8)18(1) | 29(1) | 21(1) | 1(1) | 3(1) | −1(1) | |
| C(9)32(1) | 28(1) | 22(1) | −5(1) | 8(1) | 3(1) | |
| C(10) | 33(1) | 25(1) | 28(1) | 1(1) | 4(1) | −9(1) |
| C(11) | 18(1) | 24(1) | 14(1) | −1(1) | 5(1) | 4(1) |
| C(12) | 21(1) | 18(1) | 17(1) | −2(1) | 5(1) | 0(1) |
| C(13) | 16(1) | 22(1) | 15(1) | −2(1) | 4(1) | −2(1) |
| C(14) | 17(1) | 20(1) | 16(1) | 1(1) | 7(1) | 2(1) |
| C(15) | 21(1) | 20(1) | 23(1) | −1(1) | 9(1) | −4(1) |
| C(16) | 16(1) | 26(1) | 20(1) | −3(1) | 6(1) | −3(1) |

**Table S23.** Hydrogen coordinates (×10$^4$) and isotropic displacement parameters (Å$^2$ × 10$^3$) for ester **3**.

| | $x$ | $y$ | $z$ | $U_{eq}$ |
|---|---|---|---|---|
| H(2)7490 | 125 | 2784 | 22 | |
| H(3A) | 6686 | −1859 | 3151 | 27 |
| H(3B) | 7396 | −2625 | 3208 | 27 |
| H(4A) | 7079 | −1035 | 4295 | 29 |
| H(4B) | 7470 | −2883 | 4331 | 29 |
| H(5A) | 8147 | −27 | 4806 | 26 |
| H(5B) | 8424 | −1441 | 4400 | 26 |
| H(8A) | 9179 | 717 | 4986 | 35 |
| H(8B) | 9716 | 2260 | 5087 | 35 |
| H(8C) | 9456 | 1119 | 4395 | 35 |
| H(9A) | 8108 | 4452 | 4732 | 42 |
| H(9B) | 8814 | 4231 | 5338 | 42 |
| H(9C) | 8309 | 2585 | 5124 | 42 |
| H(10A) | 9283 | 4275 | 3900 | 46 |
| H(10B) | 9390 | 5228 | 4614 | 46 |
| H(10C) | 8711 | 5498 | 3974 | 46 |
| H(12) | 5205 | 1195 | 1799 | 23 |
| H(13) | 4370 | 3363 | 1359 | 22 |
| H(15) | 5744 | 7289 | 2093 | 25 |
| H(16) | 6577 | 5114 | 2517 | 25 |

**Table S24.** Hydrogen bonds for ester **3** [Å and °].

| D–H···A | $d_{D–H}$ | $d_{H\cdots A}$ | $d_{D\cdots A}$ | $\angle_{DHA}$ |
|---|---|---|---|---|
| C(2)-H(2)...O(3)#1 | 1.00 | 2.52 | 3.2523(13) | 129.4 |
| C(12)-H(12)...O(5)#2 | 0.95 | 2.43 | 3.3506(15) | 162.5 |
| C(13)-H(13)...O(1)#3 | 0.95 | 2.54 | 3.3701(13) | 145.4 |

Symmetry transformations used to generate equivalent atoms: #1 $-x + 3/2, y - 1/2, -z + 1/2$; #2 $x, y - 1, z$; #3 $-x + 1, y, -z + 1/2$.

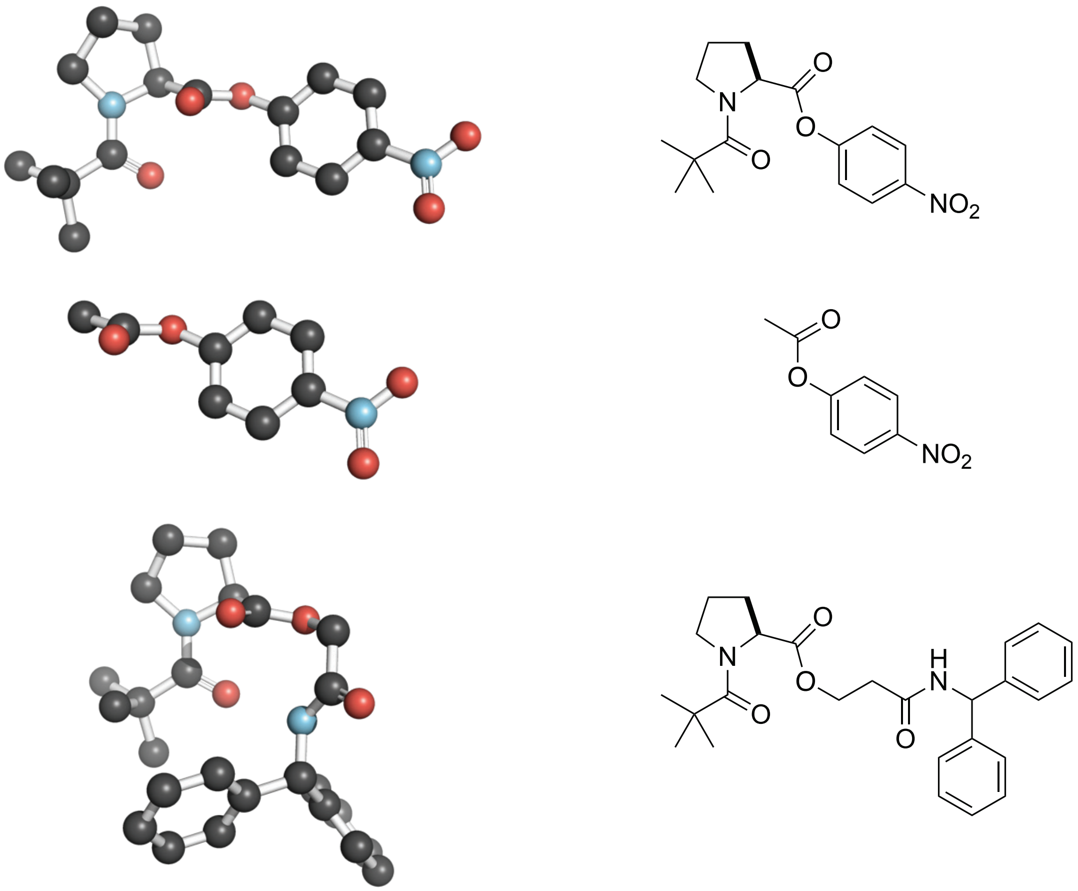

**Figure S12**. Crystal structures of ester **3** (top) and two related esters: *p*-nitrophenyl acetate (CCDC 1898137; one of two molecules in the unit cell) and PivProOBg (CCDC 196946).[21,22]

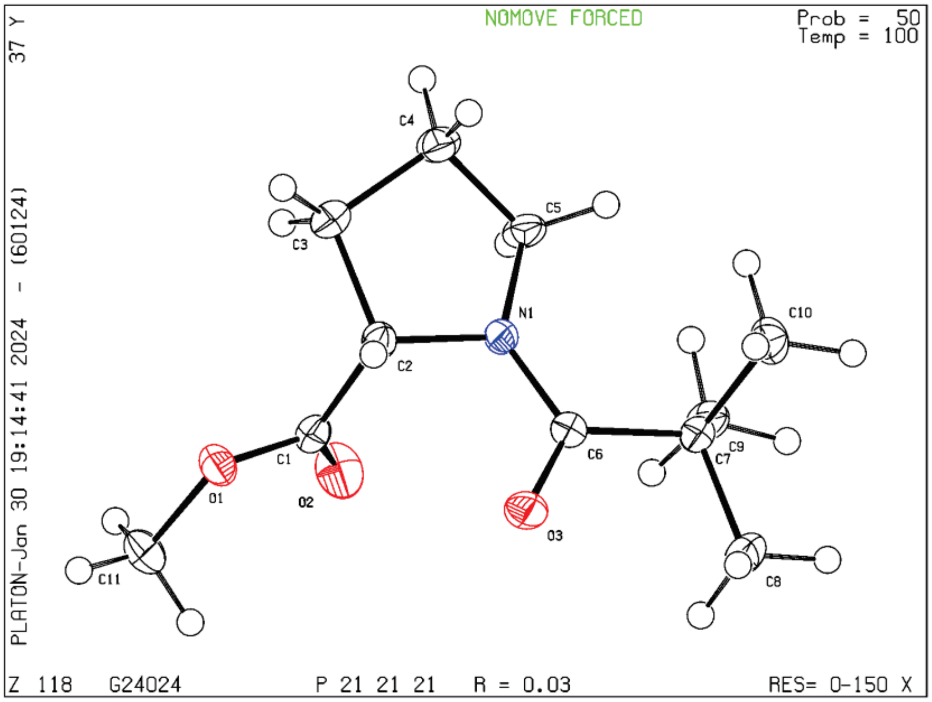


**Figure S13.** Oak Ridge thermal ellipsoid plot (ORTEP) of *N*-pivaloyl-(2*S*)-proline methyl ester from X-ray crystallography.

**Table S25.** Crystal data and structure refinement for *N*-pivaloyl-(2*S*)-proline methyl ester.

| | |
|---|---|
| CCDC number | 2350602 |
| Identification code | G24024 |
| Empirical formula | $C_{11}H_{19}NO_3$ |
| Formula weight | 213.27 |
| Temperature | 100(2) K |
| Wavelength | 1.54178 Å |
| Crystal system | Orthorhombic |
| Space group | $P2_12_12_1$ |
| Unit cell dimensions | |
| $a$ = 6.2319(2) Å | $\alpha$ = 90° |
| $b$ = 8.5204(2) Å | $\beta$ = 90° |
| $c$ = 22.3902(6) Å | $\gamma$ = 90° |
| Volume | 1188.88(6) Å$^3$ |
| $Z$ | 4 |
| Density (calculated) | 1.192 Mg/m$^3$ |
| Absorption coefficient | 0.702 mm$^{-1}$ |
| $F_{000}$ | 464 |
| Crystal size | 0.180 × 0.175 × 0.150 mm$^3$ |
| Theta range for data collection | 3.948 to 74.510° |
| Index ranges | $-7 \le h \le 7$, $-10 \le k \le 10$, $-27 \le l \le 27$ |
| Reflections collected | 78505 |
| Independent reflections | 2429 [$R_{int}$ = 0.0255] |
| Completeness to theta = 67.679° | 99.9% |
| Absorption correction | Semi-empirical from equivalents |
| Refinement method | Full-matrix least-squares on $F^2$ |
| Data/restraints/parameters | 2429/0/140 |
| Goodness-of-fit on $F^2$ | 1.089 |
| Final $R$ indices [$I > 2\sigma_I$] | $R1$ = 0.0258, w$R2$ = 0.0722 |
| $R$ indices (all data) | $R1$ = 0.0259, w$R2$ = 0.0724 |
| Absolute structure parameter | 0.030(18) |
| Extinction coefficient | n/a |
| Largest diff. peak and hole | 0.135 and −0.172 e·Å$^{-3}$ |

**Table S26.** Atomic coordinates (×10$^4$) and equivalent isotropic displacement parameters (Å$^2$ × 10$^3$) for *N*-pivaloyl-(2*S*)-proline methyl ester. $U_{eq}$ is defined as one-third of the trace of the orthogonalized $U^{ij}$ tensor.

| | *x* | *y* | *z* | $U_{eq}$ |
|---|---|---|---|---|
| O(1) | 9657(2) | 8872(1) | 7241(1) | 22(1) |
| O(2) | 7182(2) | 6982(1) | 7359(1) | 33(1) |
| O(3) | 8709(1) | 6185(1) | 6013(1) | 24(1) |
| N(1) | 5713(2) | 7524(1) | 6194(1) | 18(1) |
| C(11) | 10666(3) | 8393(2) | 7794(1) | 29(1) |
| C(1) | 7939(2) | 8044(2) | 7076(1) | 19(1) |
| C(2) | 7012(2) | 8701(1) | 6502(1) | 18(1) |
| C(3) | 5430(2) | 10043(2) | 6646(1) | 27(1) |
| C(4) | 3498(2) | 9715(2) | 6247(1) | 26(1) |
| C(5) | 3426(2) | 7922(2) | 6232(1) | 23(1) |
| C(6) | 6741(2) | 6256(2) | 5969(1) | 17(1) |
| C(7) | 5473(2) | 4931(1) | 5660(1) | 19(1) |
| C(8) | 7076(2) | 3623(2) | 5507(1) | 28(1) |
| C(9) | 3747(2) | 4250(2) | 6075(1) | 23(1) |
| C(10) | 4472(3) | 5524(2) | 5072(1) | 28(1) |

**Table S27.** Bond lengths [Å] and angles [°] for *N*-pivaloyl-(2*S*)-proline methyl ester.

| | |
|---|---|
| O(1)-C(1) | 1.3345(16) |
| O(1)-C(11) | 1.4481(15) |
| O(2)-C(1) | 1.2002(16) |
| O(3)-C(6) | 1.2317(15) |
| N(1)-C(6) | 1.3537(16) |
| N(1)-C(2) | 1.4619(15) |
| N(1)-C(5) | 1.4679(16) |
| C(11)-H(11A) | 0.9800 |
| C(11)-H(11B) | 0.9800 |
| C(11)-H(11C) | 0.9800 |
| C(1)-C(2) | 1.5165(17) |
| C(2)-C(3) | 1.5430(18) |
| C(2)-H(2) | 1.0000 |
| C(3)-C(4) | 1.5245(19) |
| C(3)-H(3A) | 0.9900 |
| C(3)-H(3B) | 0.9900 |
| C(4)-C(5) | 1.5287(17) |
| C(4)-H(4A) | 0.9900 |
| C(4)-H(4B) | 0.9900 |
| C(5)-H(5A) | 0.9900 |
| C(5)-H(5B) | 0.9900 |
| C(6)-C(7) | 1.5418(17) |
| C(7)-C(8) | 1.5349(17) |
| C(7)-C(9) | 1.5358(18) |
| C(7)-C(10) | 1.5418(18) |
| C(8)-H(8A) | 0.9800 |
| C(8)-H(8B) | 0.9800 |
| C(8)-H(8C) | 0.9800 |
| C(9)-H(9A) | 0.9800 |
| C(9)-H(9B) | 0.9800 |
| C(9)-H(9C) | 0.9800 |
| C(10)-H(10A) | 0.9800 |
| C(10)-H(10B) | 0.9800 |
| C(10)-H(10C) | 0.9800 |
| | |
| C(1)-O(1)-C(11) | 115.87(10) |
| C(6)-N(1)-C(2) | 117.49(10) |
| C(6)-N(1)-C(5) | 131.74(11) |
| C(2)-N(1)-C(5) | 110.59(10) |
| O(1)-C(11)-H(11A) | 109.5 |
| O(1)-C(11)-H(11B) | 109.5 |
| H(11A)-C(11)-H(11B) | 109.5 |
| O(1)-C(11)-H(11C) | 109.5 |
| H(11A)-C(11)-H(11C) | 109.5 |
| H(11B)-C(11)-H(11C) | 109.5 |
| O(2)-C(1)-O(1) | 124.60(12) |
| O(2)-C(1)-C(2) | 125.13(12) |
| O(1)-C(1)-C(2) | 110.18(10) |
| N(1)-C(2)-C(1) | 110.93(10) |
| N(1)-C(2)-C(3) | 104.65(10) |
| C(1)-C(2)-C(3) | 109.90(10) |
| N(1)-C(2)-H(2) | 110.4 |
| C(1)-C(2)-H(2) | 110.4 |
| C(3)-C(2)-H(2) | 110.4 |
| C(4)-C(3)-C(2) | 104.30(11) |
| C(4)-C(3)-H(3A) | 110.9 |
| C(2)-C(3)-H(3A) | 110.9 |
| C(4)-C(3)-H(3B) | 110.9 |
| C(2)-C(3)-H(3B) | 110.9 |
| H(3A)-C(3)-H(3B) | 108.9 |
| C(3)-C(4)-C(5) | 102.66(11) |
| C(3)-C(4)-H(4A) | 111.2 |
| C(5)-C(4)-H(4A) | 111.2 |
| C(3)-C(4)-H(4B) | 111.2 |
| C(5)-C(4)-H(4B) | 111.2 |
| H(4A)-C(4)-H(4B) | 109.1 |
| N(1)-C(5)-C(4) | 101.74(11) |
| N(1)-C(5)-H(5A) | 111.4 |
| C(4)-C(5)-H(5A) | 111.4 |
| N(1)-C(5)-H(5B) | 111.4 |

| | |
|---|---|
| C(4)-C(5)-H(5B) | 111.4 |
| H(5A)-C(5)-H(5B) | 109.3 |
| O(3)-C(6)-N(1) | 118.70(13) |
| O(3)-C(6)-C(7) | 120.70(12) |
| N(1)-C(6)-C(7) | 120.60(11) |
| C(8)-C(7)-C(9) | 108.43(11) |
| C(8)-C(7)-C(6) | 107.34(11) |
| C(9)-C(7)-C(6) | 111.34(10) |
| C(8)-C(7)-C(10) | 108.13(11) |
| C(9)-C(7)-C(10) | 110.94(11) |
| C(6)-C(7)-C(10) | 110.52(10) |
| C(7)-C(8)-H(8A) | 109.5 |
| C(7)-C(8)-H(8B) | 109.5 |
| H(8A)-C(8)-H(8B) | 109.5 |
| C(7)-C(8)-H(8C) | 109.5 |
| H(8A)-C(8)-H(8C) | 109.5 |
| H(8B)-C(8)-H(8C) | 109.5 |
| C(7)-C(9)-H(9A) | 109.5 |
| C(7)-C(9)-H(9B) | 109.5 |
| H(9A)-C(9)-H(9B) | 109.5 |
| C(7)-C(9)-H(9C) | 109.5 |
| H(9A)-C(9)-H(9C) | 109.5 |
| H(9B)-C(9)-H(9C) | 109.5 |
| C(7)-C(10)-H(10A) | 109.5 |
| C(7)-C(10)-H(10B) | 109.5 |
| H(10A)-C(10)-H(10B) | 109.5 |
| C(7)-C(10)-H(10C) | 109.5 |
| H(10A)-C(10)-H(10C) | 109.5 |
| H(10B)-C(10)-H(10C) | 109.5 |

**Table S28.** Anisotropic displacement parameters (Å$^2$ × 10$^3$) for *N*-pivaloyl-(2*S*)-proline methyl ester. The anisotropic displacement factor exponent takes the form: $-2\pi^2[h^2 a^{*2}U^{11} + ... + 2 h k a^* b^* U^{12}]$.

| | $U^{11}$ | $U^{22}$ | $U^{33}$ | $U^{23}$ | $U^{13}$ | $U^{12}$ |
|---|---|---|---|---|---|---|
| O(1) | 23(1) | 24(1) | 21(1) | 1(1) | −5(1) | −2(1) |
| O(2) | 41(1) | 32(1) | 26(1) | 10(1) | −4(1) | −12(1) |
| O(3) | 14(1) | 26(1) | 32(1) | −3(1) | 0(1) | 2(1) |
| N(1) | 14(1) | 18(1) | 22(1) | −3(1) | −2(1) | −1(1) |
| C(11) | 32(1) | 34(1) | 22(1) | 0(1) | −10(1) | 2(1) |
| C(1) | 21(1) | 17(1) | 19(1) | −3(1) | 2(1) | 1(1) |
| C(2) | 19(1) | 17(1) | 19(1) | −1(1) | −1(1) | −2(1) |
| C(3) | 29(1) | 18(1) | 34(1) | −4(1) | −6(1) | 4(1) |
| C(4) | 22(1) | 17(1) | 38(1) | 0(1) | −4(1) | 2(1) |
| C(5) | 15(1) | 17(1) | 36(1) | −1(1) | 1(1) | 2(1) |
| C(6) | 16(1) | 18(1) | 16(1) | 1(1) | 1(1) | 0(1) |
| C(7) | 19(1) | 16(1) | 21(1) | −2(1) | −1(1) | 0(1) |
| C(8) | 30(1) | 22(1) | 31(1) | −8(1) | 2(1) | 4(1) |
| C(9) | 22(1) | 17(1) | 32(1) | 2(1) | 1(1) | −2(1) |
| C(10) | 35(1) | 26(1) | 23(1) | −1(1) | −8(1) | −2(1) |

**Table S29.** Hydrogen coordinates (×10$^4$) and isotropic displacement parameters (Å$^2$ × 10$^3$) for *N*-pivaloyl-(2*S*)-proline methyl ester.

| | *x* | *y* | *z* | $U_{eq}$ |
|---|---|---|---|---|
| H(11A) | 9646 | 8517 | 8124 | 44 |
| H(11B) | 11931 | 9048 | 7868 | 44 |
| H(11C) | 11102 | 7291 | 7765 | 44 |
| H(2) | 8186 | 9087 | 6235 | 22 |
| H(3A) | 5017 | 10026 | 7072 | 32 |
| H(3B) | 6071 | 11076 | 6551 | 32 |
| H(4A) | 3708 | 10155 | 5842 | 31 |
| H(4B) | 2167 | 10155 | 6421 | 31 |
| H(5A) | 2767 | 7490 | 6599 | 27 |
| H(5B) | 2626 | 7535 | 5879 | 27 |
| H(8A) | 7724 | 3223 | 5875 | 42 |
| H(8B) | 8201 | 4043 | 5246 | 42 |
| H(8C) | 6325 | 2769 | 5301 | 42 |
| H(9A) | 3189 | 3273 | 5904 | 35 |
| H(9B) | 2574 | 5007 | 6118 | 35 |
| H(9C) | 4377 | 4034 | 6468 | 35 |
| H(10A) | 3882 | 4634 | 4849 | 42 |
| H(10B) | 5576 | 6045 | 4831 | 42 |
| H(10C) | 3320 | 6271 | 5162 | 42 |

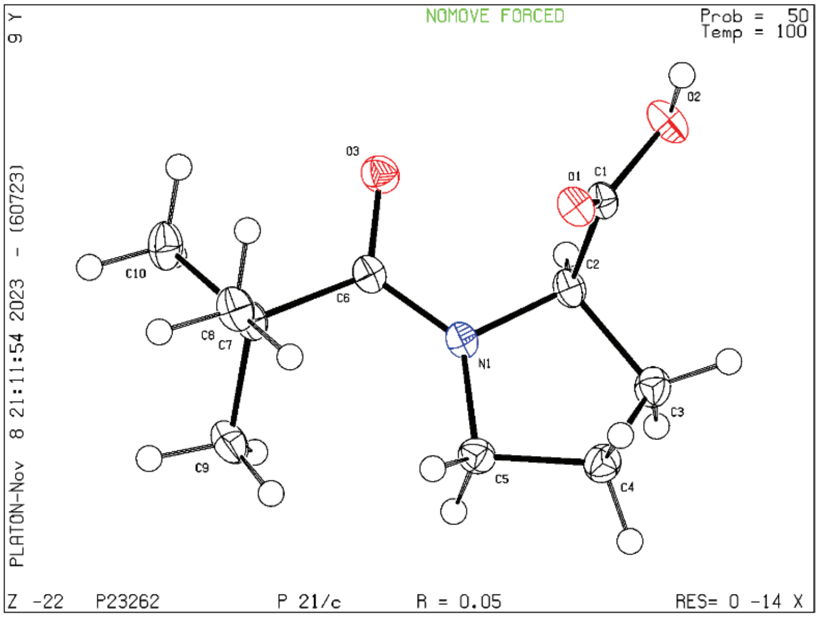


**Figure 14.** Oak Ridge thermal ellipsoid plot (ORTEP) of *N*-pivaloyl-(2*R*)-proline (**D****S2**) from X-ray crystallography.

**Table S30.** Crystal data and structure refinement for *N*-pivaloyl-(2*R*)-proline (**D S2**).

| | |
|---|---|
| CCDC number | 2350601 |
| Identification code | P23262 |
| Empirical formula | $C_{10}H_{17}NO_3$ |
| Formula weight | 199.24 |
| Temperature | 100(2) K |
| Wavelength | 0.71073 Å |
| Crystal system | Monoclinic |
| Space group | $P2_1/c$ |
| Unit cell dimensions | |
| $a$ = 16.2723(13) Å | $\alpha$ = 90° |
| $b$ = 6.3638(5) Å | $\beta$ = 98.146(3)° |
| $c$ = 10.0694(7) Å | $\gamma$ = 90° |
| Volume | 1032.20(14) $Å^3$ |
| $Z$ | 4 |
| Density (calculated) | 1.282 $Mg/m^3$ |
| Absorption coefficient | 0.094 $mm^{-1}$ |
| $F_{000}$ | 432 |
| Crystal size | 0.495 × 0.490 × 0.040 $mm^3$ |
| Theta range for data collection | 2.529 to 32.032° |
| Index ranges | $-24 \le h \le 24$, $-9 \le k \le 9$, $-15 \le l \le 14$ |
| Reflections collected | 67419 |
| Independent reflections | 3597 [$R_{int}$ = 0.0678] |
| Completeness to theta = 25.242° | 99.9% |
| Absorption correction | Semi-empirical from equivalents |
| Refinement method | Full-matrix least-squares on $F^2$ |
| Data/restraints/parameters | 3597/1/133 |
| Goodness-of-fit on $F^2$ | 1.071 |
| Final $R$ indices [$I > 2\sigma_I$] | $R1$ = 0.0502, w$R2$ = 0.1271 |
| $R$ indices (all data) | $R1$ = 0.0583, w$R2$ = 0.1347 |
| Extinction coefficient | n/a |
| Largest diff. peak and hole | 0.395 and −0.343 e·$Å^{-3}$ |

**Table S31.** Atomic coordinates (×10[4]) and equivalent isotropic displacement parameters (Å[2] × 10[3]) for acid **DS2**. $U_{eq}$ is defined as one-third of the trace of the orthogonalized $U^{ij}$ tensor.

| | *x* | *y* | *z* | $U_{eq}$ |
|---|---|---|---|---|
| O(1)4166(1) | 8751(1) | 5297(1) | 19(1) | |
| O(2)4688(1) | 8824(1) | 3347(1) | 23(1) | |
| O(3)2550(1) | 9914(1) | 3145(1) | 21(1) | |
| N(1)2752(1) | 6699(1) | 4021(1) | 17(1) | |
| C(1)4149(1) | 8206(2) | 4126(1) | 17(1) | |
| C(2)3533(1) | 6614(2) | 3454(1) | 18(1) | |
| C(3)3852(1) | 4367(2) | 3791(1) | 22(1) | |
| C(4)3521(1) | 3898(2) | 5110(1) | 22(1) | |
| C(5)2654(1) | 4861(2) | 4881(1) | 20(1) | |
| C(6)2287(1) | 8463(2) | 3784(1) | 17(1) | |
| C(7)1447(1) | 8715(2) | 4322(1) | 19(1) | |
| C(8)1640(1) | 9604(2) | 5754(1) | 23(1) | |
| C(9)926(1) | 6701(2) | 4322(1) | 25(1) | |
| C(10) | 932(1) | 10323(2) | 3419(1) | 25(1) |

**Table S32.** Bond lengths [Å] and angles [°] for acid **DS2**.

| | | | |
|---|---|---|---|
| O(1)-C(1) | 1.2252(12) | C(6)-N(1)-C(2) | 117.11(8) |
| O(2)-C(1) | 1.3160(12) | C(6)-N(1)-C(5) | 130.89(8) |
| O(2)-H(2) | 0.868(14) | C(2)-N(1)-C(5) | 111.70(8) |
| O(3)-C(6) | 1.2367(13) | O(1)-C(1)-O(2) | 124.20(9) |
| N(1)-C(6) | 1.3560(13) | O(1)-C(1)-C(2) | 122.83(9) |
| N(1)-C(2) | 1.4659(13) | O(2)-C(1)-C(2) | 112.84(8) |
| N(1)-C(5) | 1.4774(13) | N(1)-C(2)-C(1) | 110.89(8) |
| C(1)-C(2) | 1.5160(14) | N(1)-C(2)-C(3) | 103.43(8) |
| C(2)-C(3) | 1.5422(15) | C(1)-C(2)-C(3) | 109.92(8) |
| C(2)-H(2A) | 1.0000 | N(1)-C(2)-H(2A) | 110.8 |
| C(3)-C(4) | 1.5313(16) | C(1)-C(2)-H(2A) | 110.8 |
| C(3)-H(3A) | 0.9900 | C(3)-C(2)-H(2A) | 110.8 |
| C(3)-H(3B) | 0.9900 | C(4)-C(3)-C(2) | 102.89(8) |
| C(4)-C(5) | 1.5256(15) | C(4)-C(3)-H(3A) | 111.2 |
| C(4)-H(4A) | 0.9900 | C(2)-C(3)-H(3A) | 111.2 |
| C(4)-H(4B) | 0.9900 | C(4)-C(3)-H(3B) | 111.2 |
| C(5)-H(5A) | 0.9900 | C(2)-C(3)-H(3B) | 111.2 |
| C(5)-H(5B) | 0.9900 | H(3A)-C(3)-H(3B) | 109.1 |
| C(6)-C(7) | 1.5478(14) | C(5)-C(4)-C(3) | 102.78(8) |
| C(7)-C(10) | 1.5355(15) | C(5)-C(4)-H(4A) | 111.2 |
| C(7)-C(9) | 1.5367(16) | C(3)-C(4)-H(4A) | 111.2 |
| C(7)-C(8) | 1.5390(14) | C(5)-C(4)-H(4B) | 111.2 |
| C(8)-H(8A) | 0.9800 | C(3)-C(4)-H(4B) | 111.2 |
| C(8)-H(8B) | 0.9800 | H(4A)-C(4)-H(4B) | 109.1 |
| C(8)-H(8C) | 0.9800 | N(1)-C(5)-C(4) | 103.47(8) |
| C(9)-H(9A) | 0.9800 | N(1)-C(5)-H(5A) | 111.1 |
| C(9)-H(9B) | 0.9800 | C(4)-C(5)-H(5A) | 111.1 |
| C(9)-H(9C) | 0.9800 | N(1)-C(5)-H(5B) | 111.1 |
| C(10)-H(10A) | 0.9800 | C(4)-C(5)-H(5B) | 111.1 |
| C(10)-H(10B) | 0.9800 | H(5A)-C(5)-H(5B) | 109.0 |
| C(10)-H(10C) | 0.9800 | O(3)-C(6)-N(1) | 118.89(9) |
| | | O(3)-C(6)-C(7) | 119.55(9) |
| C(1)-O(2)-H(2) | 106.7(12) | N(1)-C(6)-C(7) | 121.54(9) |
| C(10)-C(7)-C(9) | 107.57(9) | | |
| C(10)-C(7)-C(8) | 109.36(9) | | |
| C(9)-C(7)-C(8) | 110.22(9) | | |
| C(10)-C(7)-C(6) | 107.23(8) | | |

| | |
|---|---|
| C(9)-C(7)-C(6) | 115.30(9) |
| C(8)-C(7)-C(6) | 107.04(8) |
| C(7)-C(8)-H(8A) | 109.5 |
| C(7)-C(8)-H(8B) | 109.5 |
| H(8A)-C(8)-H(8B) | 109.5 |
| C(7)-C(8)-H(8C) | 109.5 |
| H(8A)-C(8)-H(8C) | 109.5 |
| H(8B)-C(8)-H(8C) | 109.5 |
| C(7)-C(9)-H(9A) | 109.5 |
| C(7)-C(9)-H(9B) | 109.5 |
| H(9A)-C(9)-H(9B) | 109.5 |
| C(7)-C(9)-H(9C) | 109.5 |
| H(9A)-C(9)-H(9C) | 109.5 |
| H(9B)-C(9)-H(9C) | 109.5 |
| C(7)-C(10)-H(10A) | 109.5 |
| C(7)-C(10)-H(10B) | 109.5 |
| H(10A)-C(10)-H(10B) | 109.5 |
| C(7)-C(10)-H(10C) | 109.5 |
| H(10A)-C(10)-H(10C) | 109.5 |
| H(10B)-C(10)-H(10C) | 109.5 |

**Table S33.** Anisotropic displacement parameters (Å$^2$ × 10$^3$) for acid **DS2**. The anisotropic displacement factor exponent takes the form: $-2\pi^2[h^2 a^{*2}U^{11} + ... + 2\, h\, k\, a^*\, b^*\, U^{12}]$.

| | $U^{11}$ | $U^{22}$ | $U^{33}$ | $U^{23}$ | $U^{13}$ | $U^{12}$ |
|---|---|---|---|---|---|---|
| O(1)18(1) | 25(1) | 15(1) | −2(1) | 2(1) | −2(1) | |
| O(2)18(1) | 33(1) | 17(1) | −4(1) | 5(1) | −7(1) | |
| O(3)21(1) | 24(1) | 19(1) | 3(1) | 4(1) | 0(1) | |
| N(1)14(1) | 21(1) | 16(1) | 1(1) | 2(1) | 0(1) | |
| C(1)14(1) | 21(1) | 16(1) | 1(1) | 2(1) | 1(1) | |
| C(2)14(1) | 23(1) | 15(1) | −2(1) | 2(1) | 0(1) | |
| C(3)20(1) | 22(1) | 23(1) | −4(1) | 4(1) | 1(1) | |
| C(4)21(1) | 21(1) | 22(1) | 1(1) | 1(1) | 2(1) | |
| C(5)20(1) | 21(1) | 18(1) | 1(1) | 3(1) | −1(1) | |
| C(6)15(1) | 22(1) | 13(1) | −1(1) | 1(1) | −1(1) | |
| C(7)15(1) | 24(1) | 17(1) | −1(1) | 2(1) | 1(1) | |
| C(8)21(1) | 31(1) | 17(1) | −2(1) | 4(1) | 0(1) | |
| C(9)17(1) | 30(1) | 29(1) | −4(1) | 5(1) | −4(1) | |
| C(10) | 18(1) | 35(1) | 23(1) | 2(1) | −1(1) | 6(1) |

**Table S34.** Hydrogen coordinates (×$10^4$) and isotropic displacement parameters (Å$^2$ × $10^3$) for acid **D S2**.

| | $x$ | $y$ | $z$ | $U_{eq}$ |
|---|---|---|---|---|
| H(2)5012(11) | 9730(30) | 3805(17) | 34 | |
| H(2A) | 3432 | 6839 | 2462 | 21 |
| H(3A) | 3627 | 3363 | 3081 | 26 |
| H(3B) | 4466 | 4313 | 3910 | 26 |
| H(4A) | 3496 | 2366 | 5271 | 26 |
| H(4B) | 3871 | 4566 | 5880 | 26 |
| H(5A) | 2244 | 3856 | 4422 | 24 |
| H(5B) | 2476 | 5296 | 5740 | 24 |
| H(8A) | 1958 | 10909 | 5737 | 34 |
| H(8B) | 1965 | 8577 | 6333 | 34 |
| H(8C) | 1119 | 9892 | 6104 | 34 |
| H(9A) | 1180 | 5763 | 5038 | 37 |
| H(9B) | 906 | 5992 | 3454 | 37 |
| H(9C) | 362 | 7061 | 4475 | 37 |
| H(10A) | 422 | 10644 | 3791 | 38 |
| H(10B) | 791 | 9738 | 2516 | 38 |
| H(10C) | 1257 | 11613 | 3375 | 38 |

**Table S35.** Hydrogen bonds for acid **DS2** [Å and °].

| D–H···A | $d_{D–H}$ | $d_{H···A}$ | $d_{D···A}$ | $\angle_{DHA}$ |
|---|---|---|---|---|
| O(2)-H(2)...O(1)#1 | 0.868(14) | 1.788(14) | 2.6457(11) | 169.0(18) |
| C(4)-H(4A)...O(1)#2 | 0.99 | 2.54 | 3.4361(14) | 149.8 |
| C(5)-H(5B)...O(3)#3 | 0.99 | 2.41 | 3.3179(13) | 152.0 |

Symmetry transformations used to generate equivalent atoms: #1 $-x + 1$, $-y + 2$, $-z + 1$; #2 $x$, $y - 1$, $z$; #3 $x$, $-y + 3/2$, $z + 1/2$.

**NMR Spectra**

*N*-Formyl-(2*S*)-proline (**S1**)
$^{1}$H NMR ($CDCl_3$, 500 MHz)

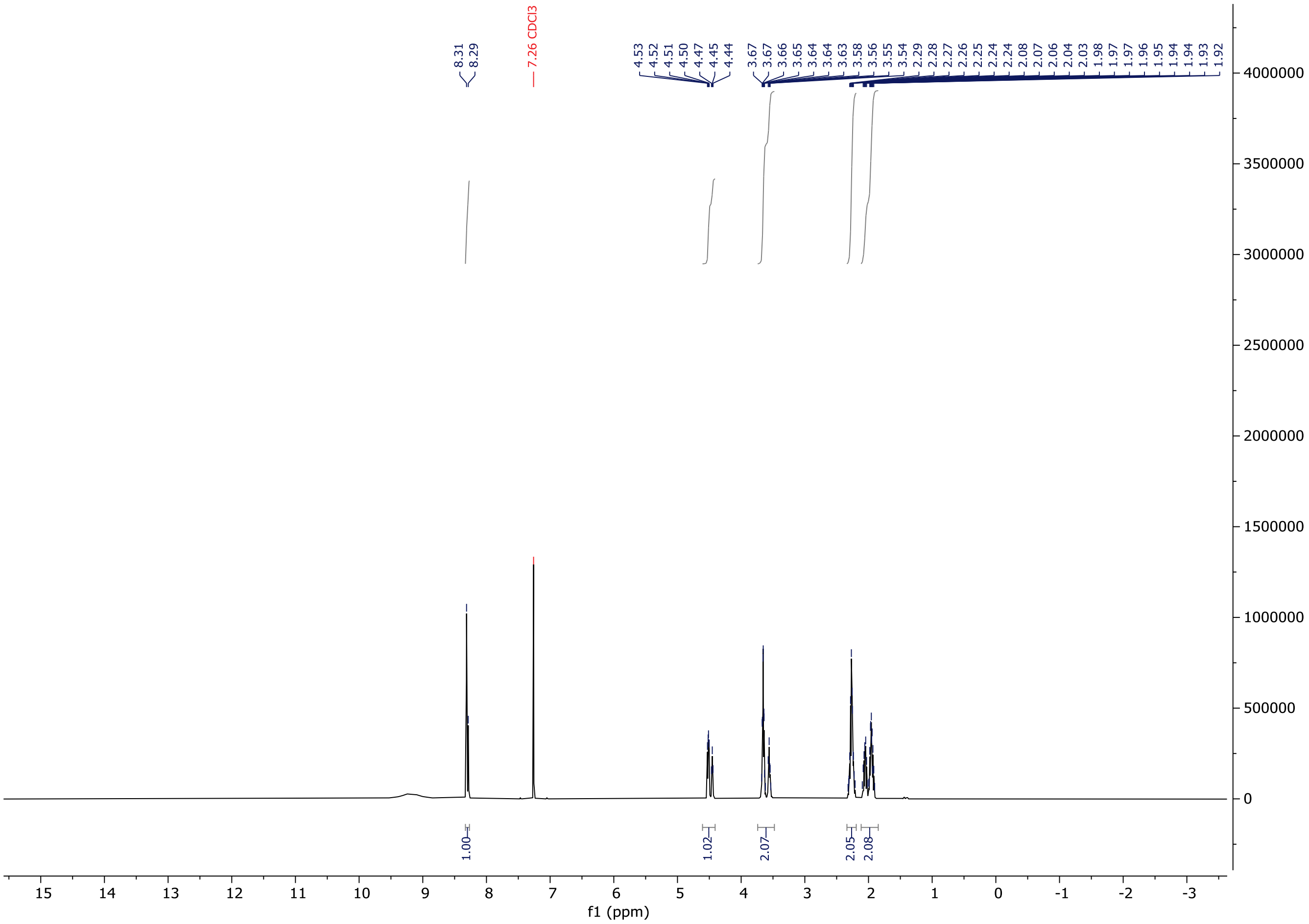

*N*-Formyl-(2*S*)-proline (**S1**)
$^{13}$C NMR ($CDCl_3$, 126 MHz)

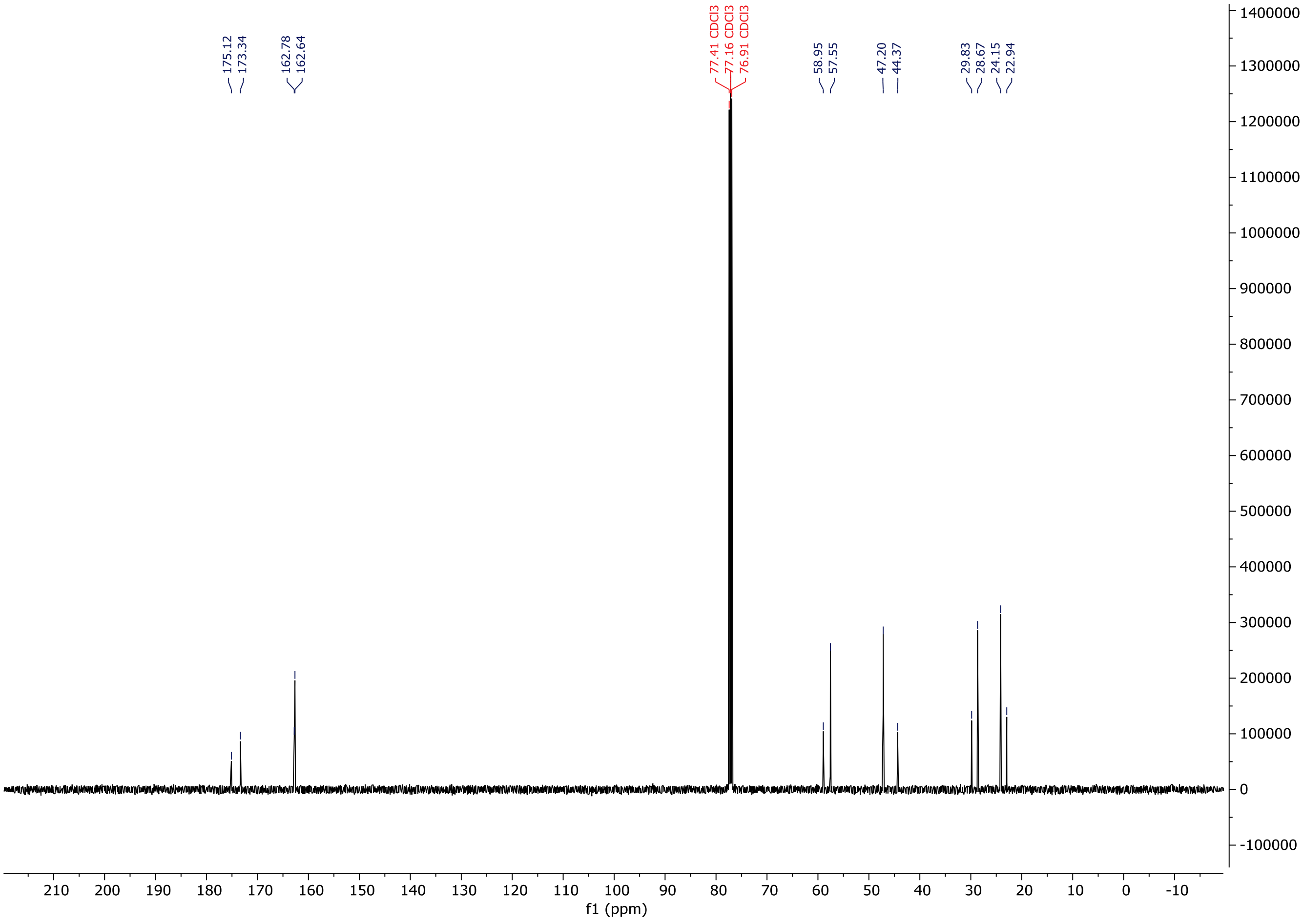

*N*-Pivaloyl-(2*S*)-proline (**S2**)
$^{1}$H NMR ($CDCl_3$, 500 MHz)

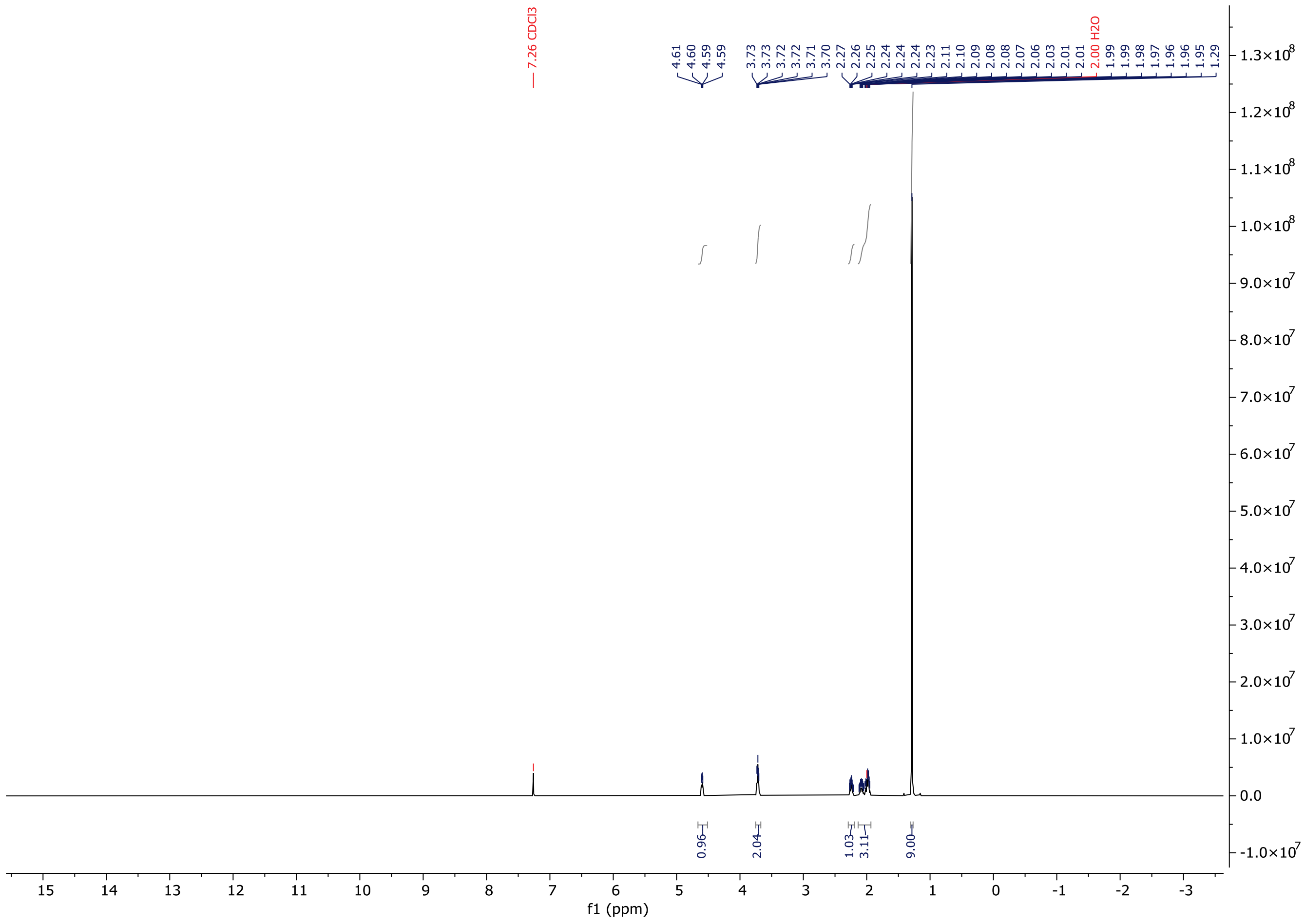

*N*-Pivaloyl-(2*S*)-proline (**S2**)
$^{13}C$ NMR ($CDCl_3$, 126 MHz)

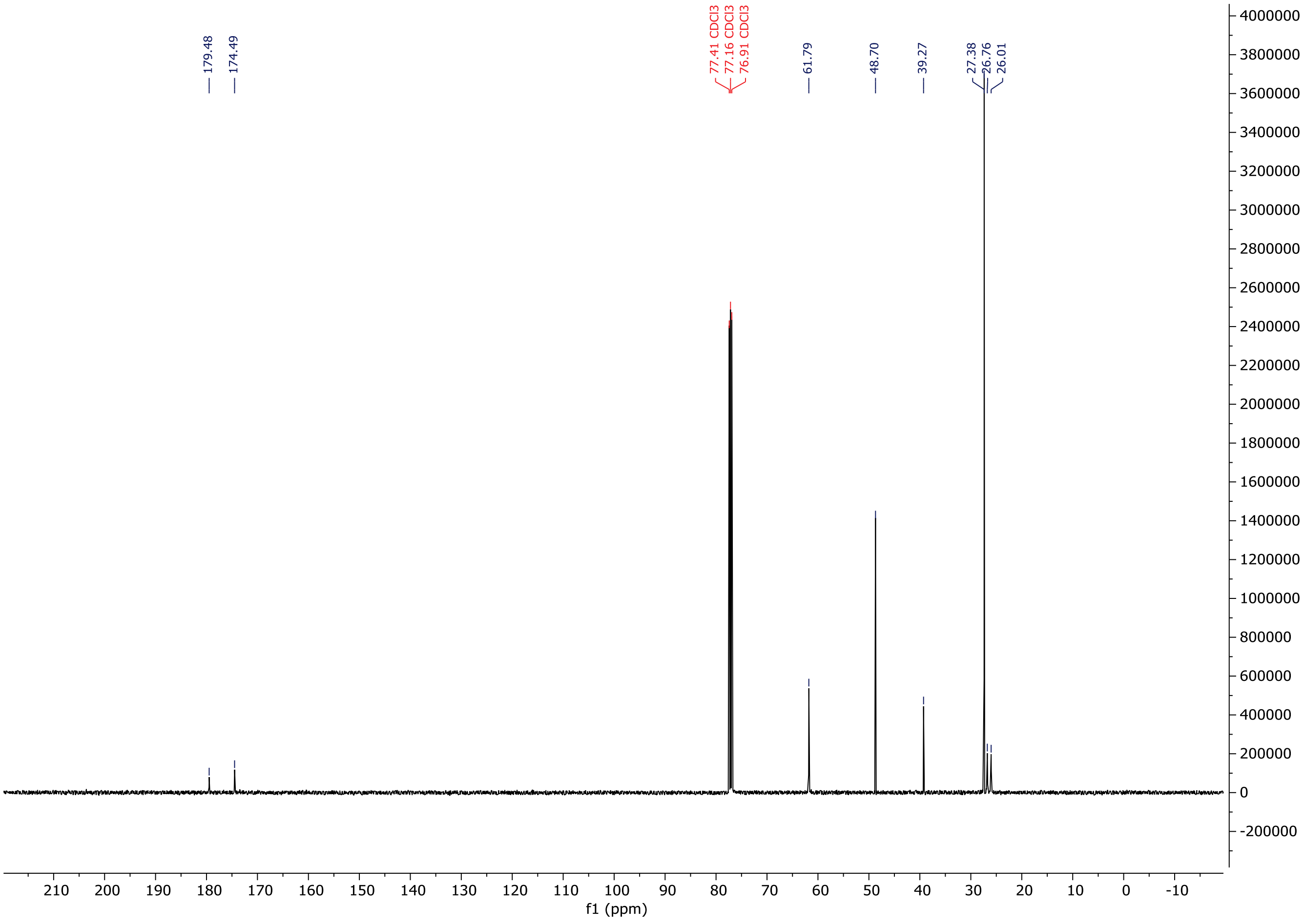

*N*-Formyl-(2*S*)-proline *p*-nitrophenyl ester (**1**)
$^{1}$H NMR ($CDCl_3$, 500 MHz)

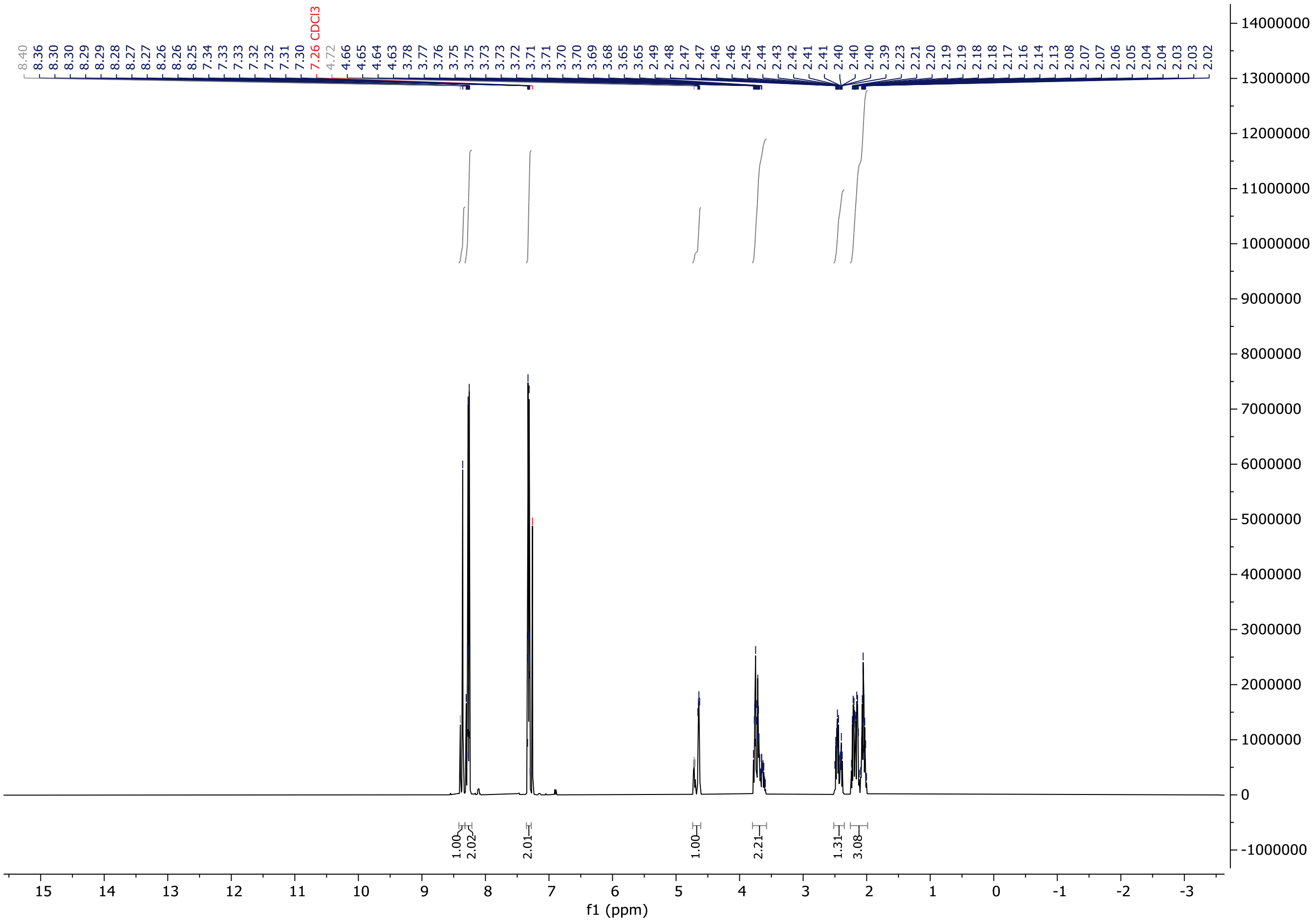

*N*-Formyl-(2*S*)-proline *p*-nitrophenyl ester (**1**)
$^{13}$C NMR ($CDCl_3$, 126 MHz)

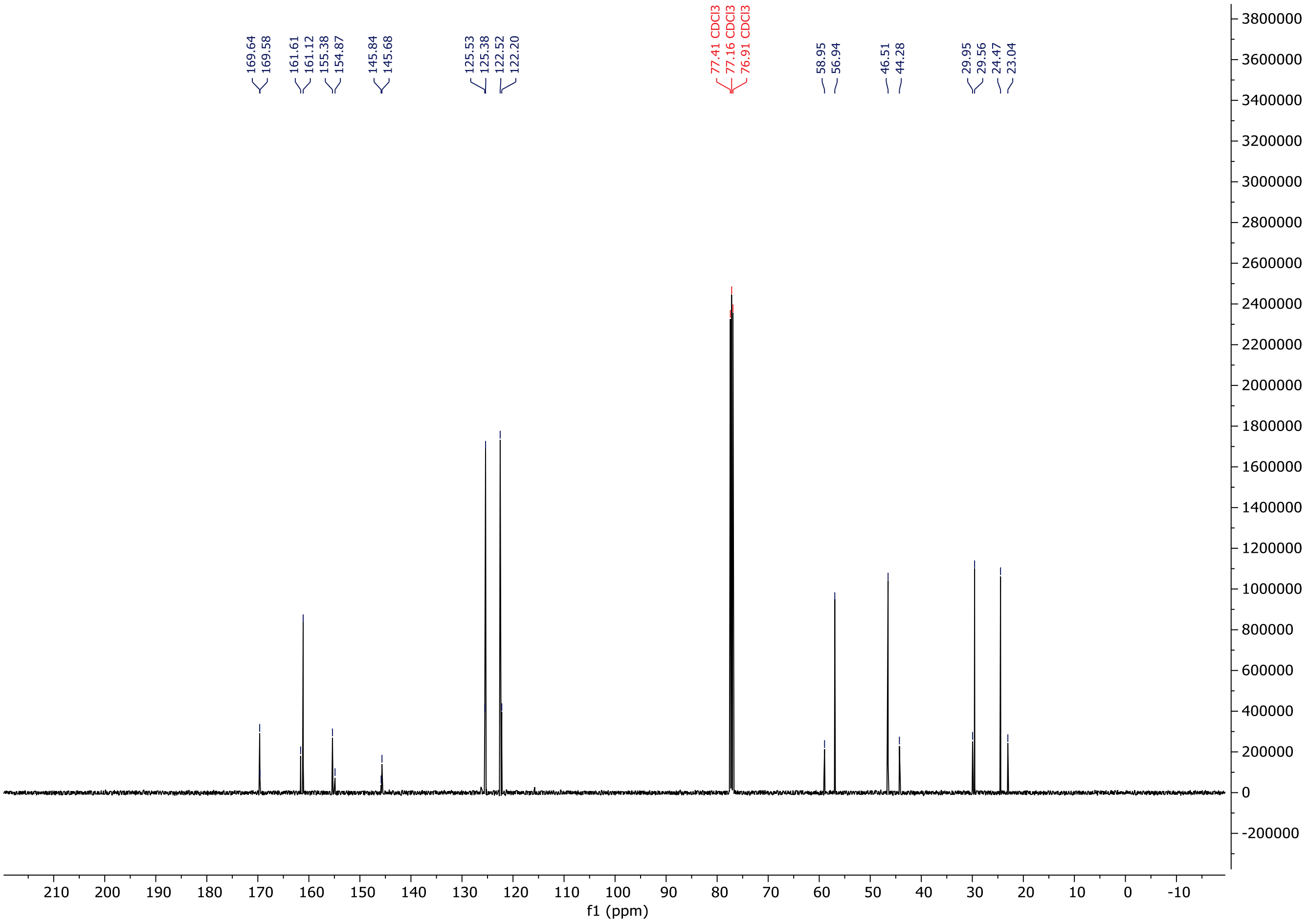

*N*-Acetyl-(2*S*)-proline *p*-nitrophenyl ester (**2**)
$^{1}$H NMR ($CDCl_3$, 500 MHz)

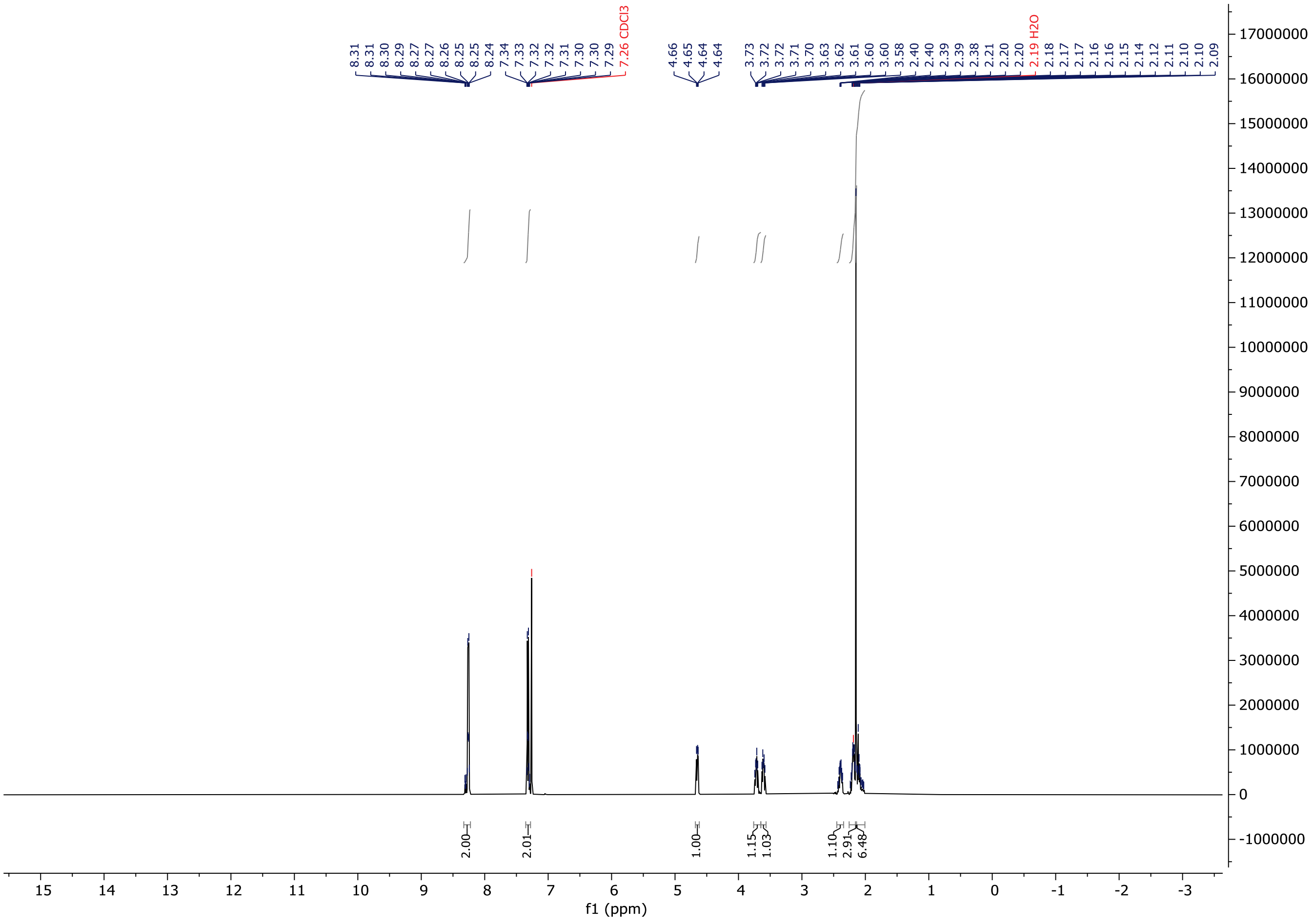

*N*-Acetyl-(2*S*)-proline *p*-nitrophenyl ester (**2**)
$^{13}$C NMR ($CDCl_3$, 126 MHz)

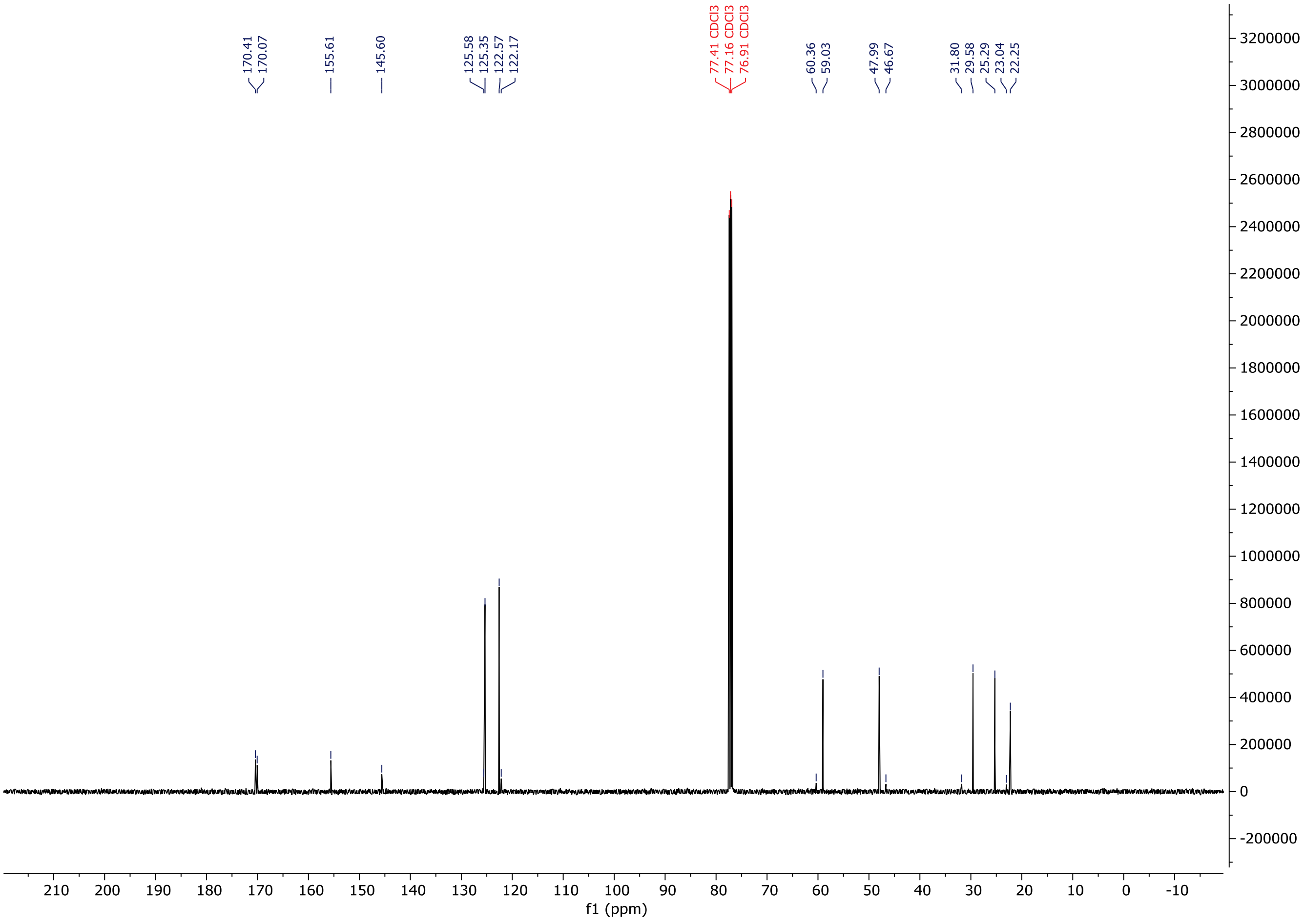

*N*-Pivaloyl-(2*S*)-proline *p*-nitrophenyl ester (**3**)
$^1$H NMR ($CDCl_3$, 500 MHz)

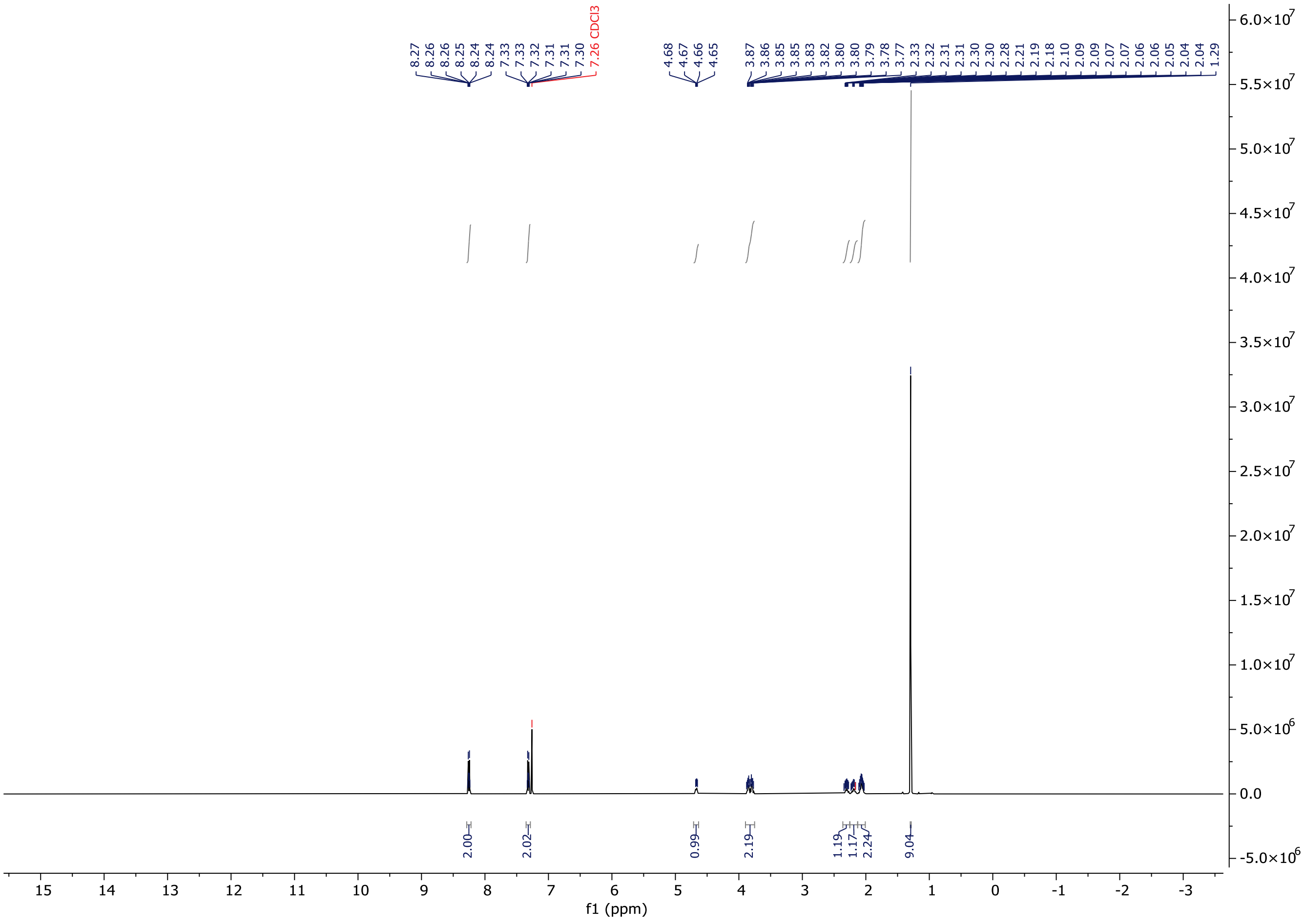

*N*-Pivaloyl-(2*S*)-proline *p*-nitrophenyl ester (**3**)
$^{13}C$ NMR ($CDCl_3$, 126 MHz)

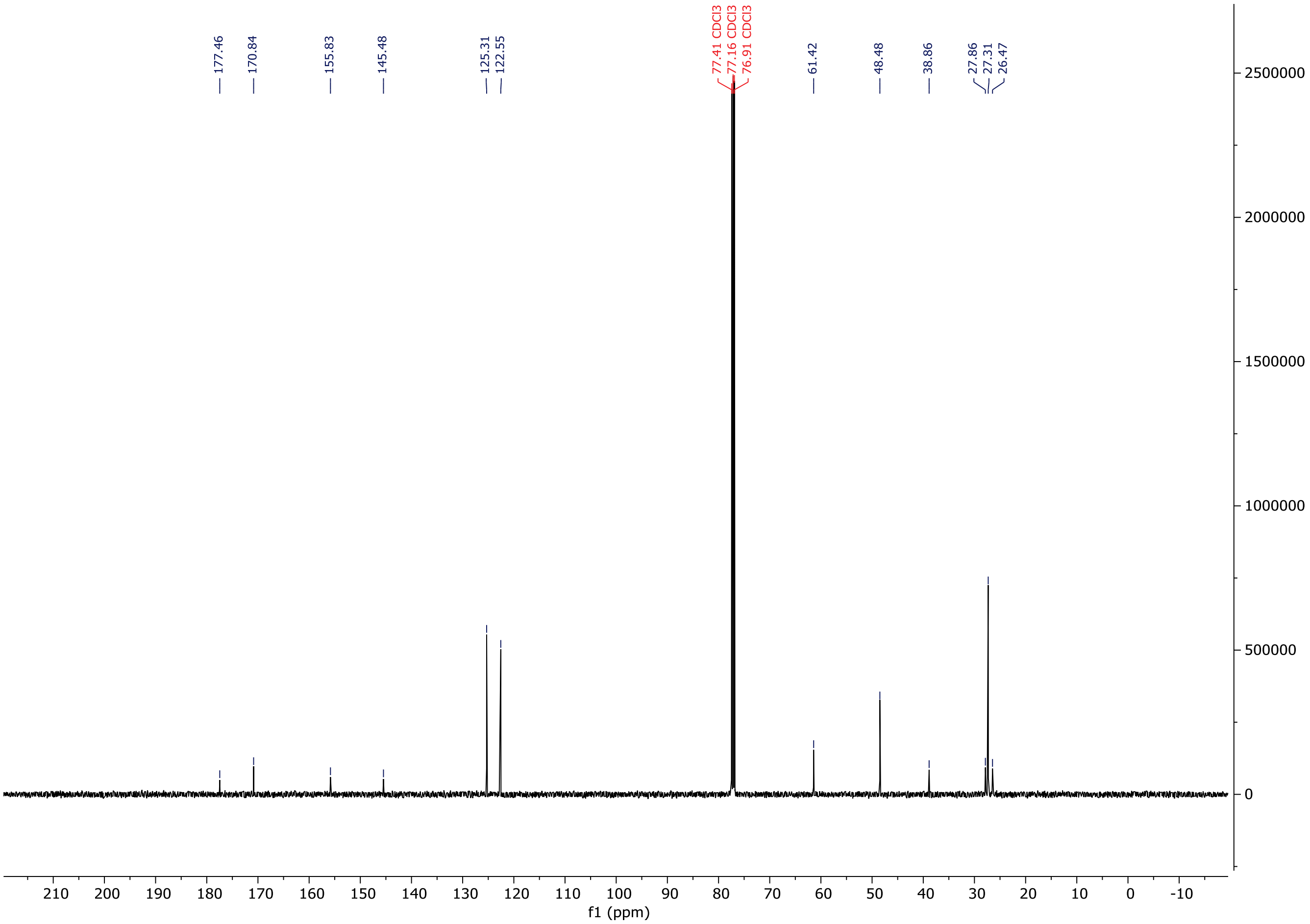

*N*-Formyl-(2*S*)-proline methyl ester
$^{1}$H NMR (dPBS, 500 MHz)

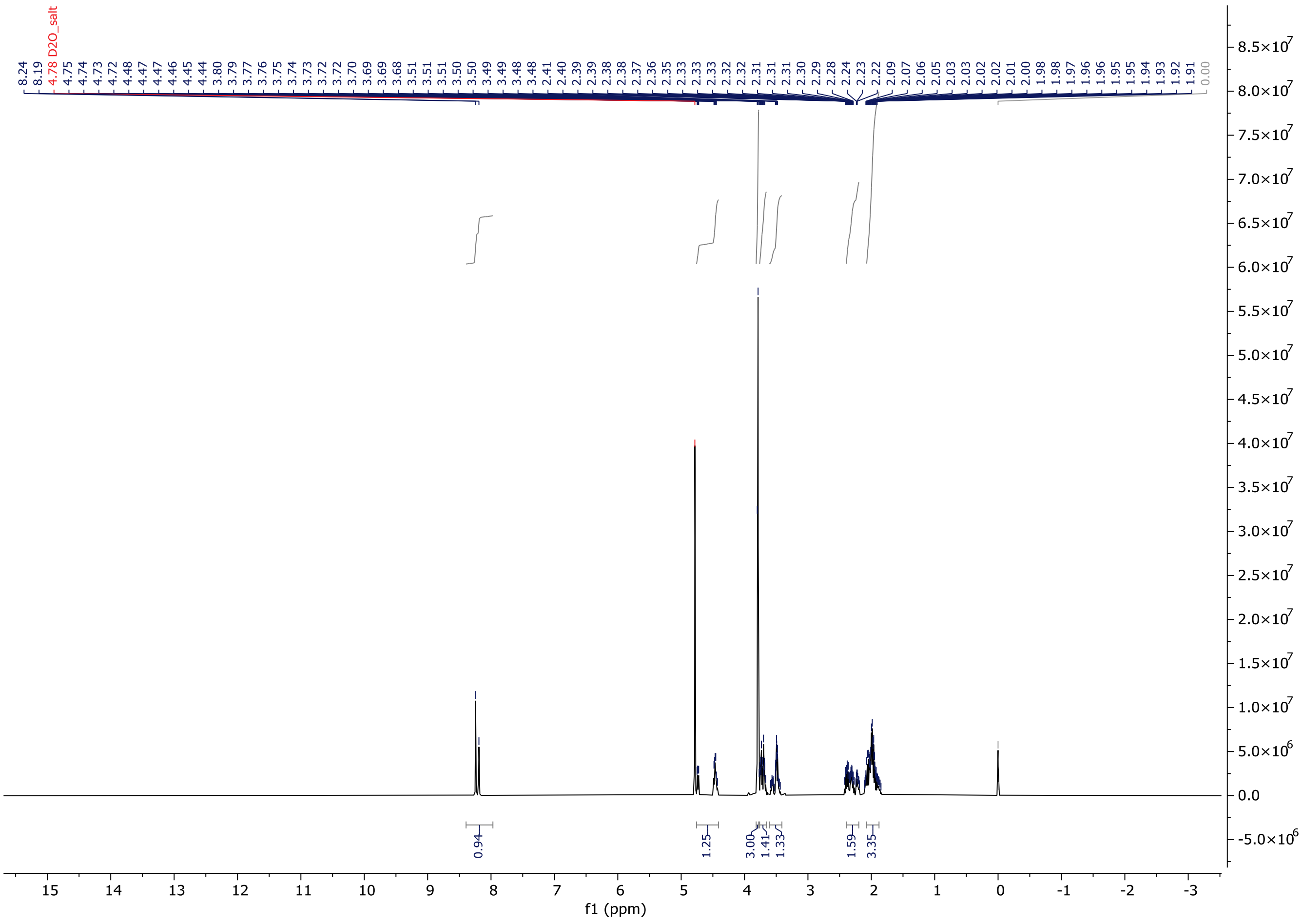

*N*-Formyl-(2*S*)-proline methyl ester
$^{13}$C NMR (dPBS, 126 MHz)

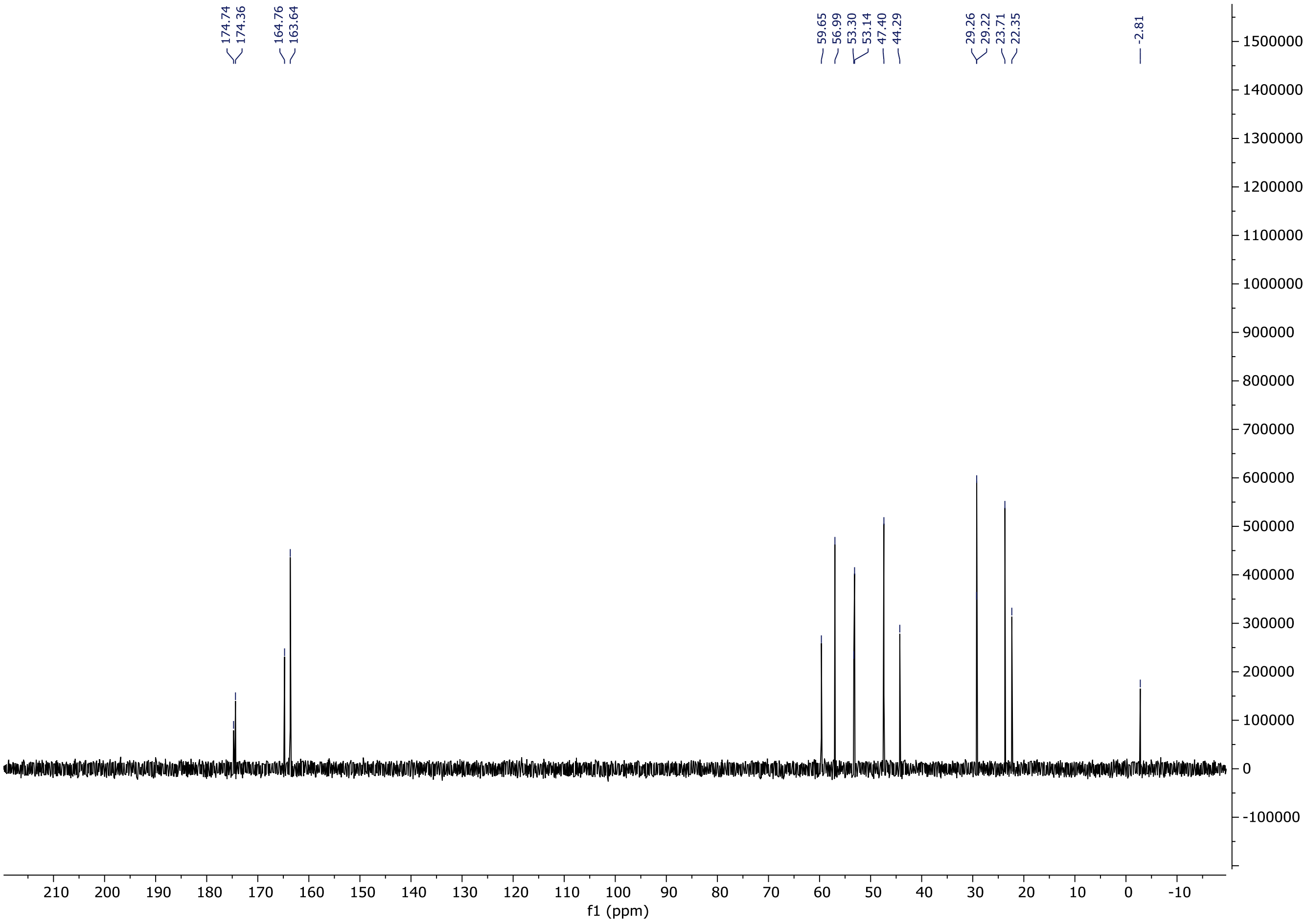

*N*-Pivaloyl-(2*S*)-proline methyl ester
$^{1}$H NMR (dPBS, 500 MHz)

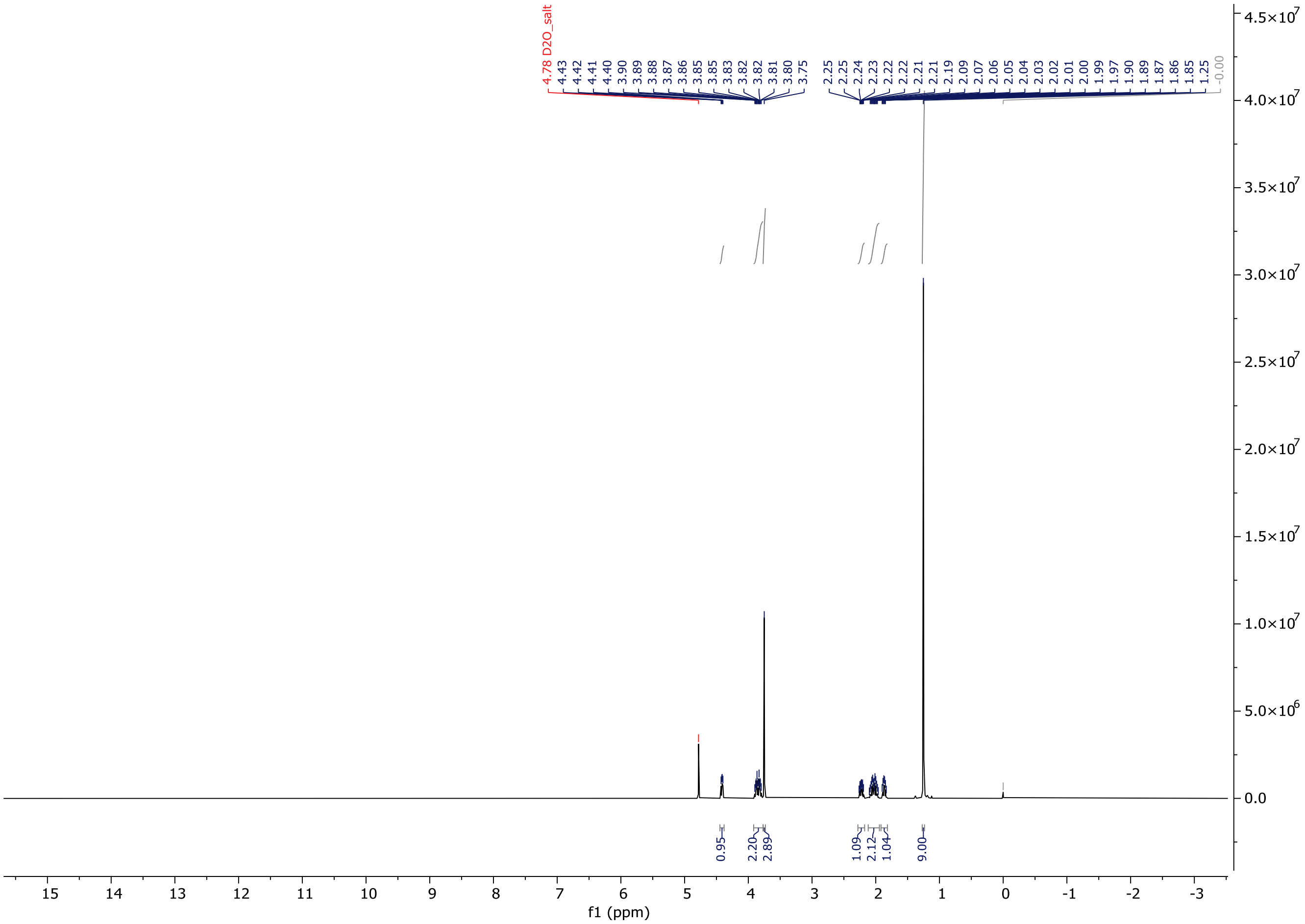

*N*-Pivaloyl-(2*S*)-proline methyl ester
$^{13}$C NMR (dPBS, 126 MHz)

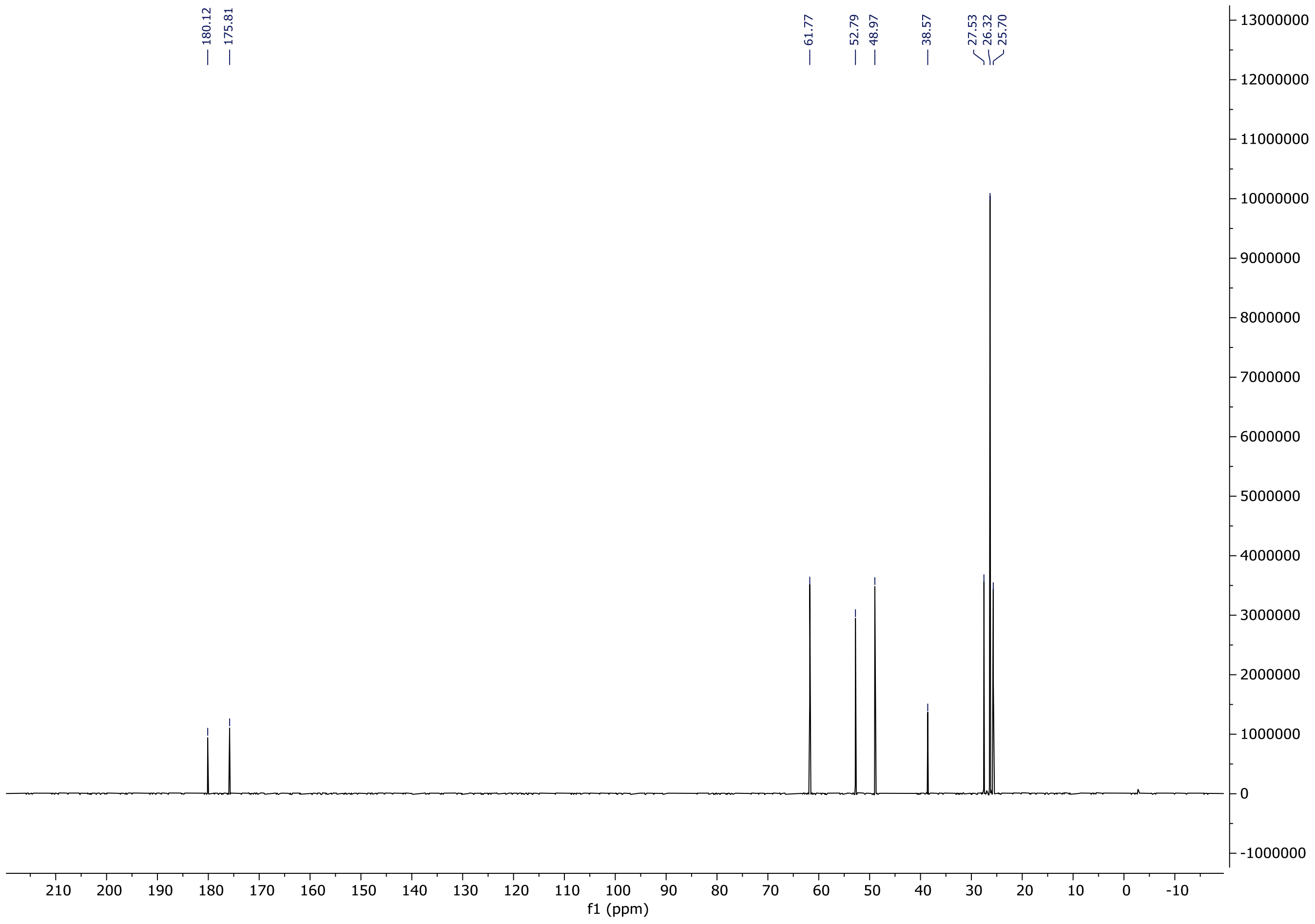

**FTIR Spectra**

Esters **1**–**3** (Film)

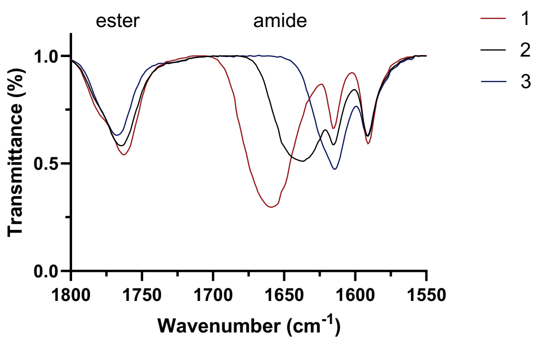

## References

(1) Hinderaker, M. P.; Raines, R. T. An electronic effect on protein structure. *Protein Sci.* **2002**, *12*, 1188–1194.

(2) Feng, R.; Wang, B.; Liu, Y.; Liu, Z.; Zhang, Y. Efficient synthesis of cis-3-substituted prolines by bidentate-assisted palladium catalysis. *Eur. J. Org. Chem.* **2015**, *7*, 142–151.

(3) Hodges, J. A.; Raines, R. T. Energetics of an n→π* interaction that impacts protein structure. *Org. Lett.* **2006**, *8*, 4695–4697.

(4) Suchý, M.; Elmehriki, A. A. H.; Hudson, R. H. E. A remarkably simple protocol for the *N*-formylation of amino acid esters and primary amines. *Org. Lett.* **2011**, *13*, 3952–3955.

(5) Reddy, D. N.; Thirupathi, R.; Tumminakattia, S.; Prabhakaran, E. N. A method for stabilizing the cis prolyl peptide bond: Influence of an unusual n→π* interaction in 1,3-oxazine and 1,3-thiazine containing peptidomimetics. *Tetrahedron Lett.* **2012**, *53*, 4413–4417.

(6) Zhao, Y.; Truhlar, D. G. Density functionals with broad applicability in chemistry. *Acc. Chem. Res.* **2008**, *41*, 157–167.

(7) Zhao, Y.; Truhlar, D. G. The M06 suite of density functionals for main group thermochemistry, thermochemical kinetics, noncovalent interactions, excited states, and transition elements: two new functionals and systematic testing of four M06-class functionals and 12 other functionals. *Theor. Chem. Account.* **2008**, *120*, 215–241.

(8) Glendening, E. D.; J, K. B.; Reed, A. E.; Carpenter, J. E.; Bohmann, J. A.; Morales, C. M.; Karafiloglou, P.; Landis, C. R.; Weinhold, F. *NBO 7.0*, University of Wisconsin, Madison: Theoretical Chemistry Institute, 2018.

(9) Frisch, M. J.; Trucks, G. W.; Schlegel, H. B.; Scuseria, G. E.; Robb, M. A.; Cheeseman, J. R.; Scalmani, G.; Barone, V.; Petersson, G. A.; Nakatsuji, H.; Li, X.; Caricato, M.; Marenich, A. V.; Bloino, J.; Janesko, B. G.; Gomperts, R.; Mennucci, B.; Hratchian, H. P.; Ortiz, J. V.; Izmaylov, A. F.; Sonnenberg, J. L.; Williams; Ding, F.; Lipparini, F.; Egidi, F.; Goings, J.; Peng, B.; Petrone, A.; Henderson, T.; Ranasinghe, D.; Zakrzewski, V. G.; Gao, J.; Rega, N.; Zheng, G.; Liang, W.; Hada, M.; Ehara, M.; Toyota, K.; Fukuda, R.; Hasegawa, J.; Ishida, M.; Nakajima, T.; Honda, Y.; Kitao, O.; Nakai, H.; Vreven, T.; Throssell, K.; Montgomery, J. A., Jr.; Peralta, J. E.; Ogliaro, F.; Bearpark, M. J.; Heyd, J. J.; Brothers, E. N.; Kudin, K. N.; Staroverov, V. N.; Keith, T. A.; Kobayashi, R.; Normand, J.; Raghavachari, K.; Rendell, A. P.; Burant, J. C.; Iyengar, S. S.; Tomasi, J.; Cossi, M.; Millam, J. M.; Klene, M.; Adamo, C.; Cammi, R.; Ochterski, J. W.; Martin, R. L.; Morokuma, K.; Farkas, O.; Foresman, J. B.; Fox, D. J. *Gaussian 16 Rev. C.01*, Wallingford, CT, 2019.

(10) Grimme, S. Exploration of chemical compound, conformer, and reaction space with meta-dynamics simulations based on tight-binding quantum chemical calculations. *J. Chem. Theory Comput.* **2019**, *15*, 2847–2862.

(11) Pracht, P.; Bohle, F.; Grimme, S. Automated exploration of the low-energy chemical space with fast quantum chemical methods. *Phys. Chem. Chem. Phys.* **2020**, *22*, 7169–7192.

(12) Frisch, M. J.; Pople, J. A.; Binkley, J. S. Self-consistent molecular orbital methods. 25. Supplementary functions for Gaussian basis sets. *J. Chem. Phys.* **1984**, *80*, 3265–3269.

(13) Adamo, C.; Barone, V. Exchange functionals with improved long-range behavior and adiabatic connection methods without adjustable parameters: The *m*PW and *m*PW1PW models. *J. Chem. Phys.* **1998**, *108*, 664–675.

(14) Marenich, A. V.; Cramer, C. J.; Truhlar, D. G. Universal solvation model based on solute electron density and on a continuum model of the solvent defined by the bulk dielectric constant and atomic surface tensions. *J. Phys. Chem. B* **2009**, *113*, 6378–6396.

(15) Wolinski, K.; Hinton, J. F.; Pulay, P. Efficient implementation of the gauge-independent atomic orbital method for NMR chemical shift calculations. *J. Am. Chem. Soc.* **1990**, *112*, 8251–8260.

(16) Cheeseman, J. R.; Trucks, G. W.; Keith, T. A.; Frisch, M. J. A comparison of models for calculating nuclear magnetic resonance shielding tensors. *J. Chem. Phys.* **1996**, *104*, 5497–5509.

(17) Bagno, A.; Saielli, G. Addressing the stereochemistry of complex organic molecules by density functional theory-NMR. *Wiley Interdiscip. Rev. Comput. Mol. Sci.* **2015**, *5*, 228–240.

(18) de Albuquerque, A. C. F.; Ribeiro, D. J.; de Amorim, M. B. Structural determination of complex natural products by quantum mechanical calculations of $^{13}C$ NMR chemical shifts: Development of a parameterized protocol for terpenes. *J. Mol. Model.* **2016**, *22*, 1–7.

(19) CHESHIRE: Chemical Shift Repository with Coupling Constants Added Too. 2019, http://cheshirenmr.info/index.htm (accessed December 29, 2023).

(20) Álvarez-Moreno, M.; de Graaf, C.; López, N.; Maseras, F.; Poblet, J. M.; Bo, C. Managing the computational chemistry big data problem: The ioChem-BD platform. *J. Chem. Inf. Model.* **2015**, *55*, 95–103.

(21) Crisma, M.; Formaggio, F.; Ruzza, P.; Calderan, A.; Elardo, S.; Borin, G.; Toniolo, C., CCDC 196946. Experimental Crystal Structure Determination, 2004.

(22) Oliver, A., CCDC 1898137. Experimental Crystal Structure Determination, 2019.
